# Association Between Brain Structure and Alcohol Use Behaviors in Adults: A Mendelian Randomization and Multiomics Study

**DOI:** 10.1001/jamapsychiatry.2022.2196

**Published:** 2022-08-10

**Authors:** Lucas A. Mavromatis, Daniel B. Rosoff, Renata B. Cupertino, Hugh Garavan, Scott Mackey, Falk W. Lohoff

**Affiliations:** 1 Section on Clinical Genomics and Experimental Therapeutics, National Institute on Alcohol Abuse and Alcoholism, National Institutes of Health, Bethesda, Maryland; 2 National Institutes of Health–Oxford-Cambridge Scholars Program; Nuffield Department of Population Health, University of Oxford, Oxford, United Kingdom; 3 Department of Psychiatry, University of Vermont College of Medicine, Burlington

## Abstract

**Importance:**

Past studies have identified associations between brain macrostructure and alcohol use behaviors. However, identifying directional associations between these phenotypes is difficult due to the limitations of observational studies.

**Objective:**

To use mendelian randomization (MR) to identify directional associations between brain structure and alcohol use and elucidate the transcriptomic and cellular underpinnings of identified associations.

**Design, Setting, and Participants:**

The main source data comprised summary statistics from population-based and case-control genome-wide association studies (GWAS) of neuroimaging, behavioral, and clinical phenotypes (N = 763 874). Using these data, bidirectional and multivariable MR was performed analyzing associations between brain macrostructure and alcohol use. Downstream transcriptome-wide association studies (TWAS) and cell-type enrichment analyses investigated the biology underlying identified associations. The study approach was data driven and did not test any a priori hypotheses. Data were analyzed August 2021 to May 2022.

**Main Outcomes and Measures:**

Brain structure phenotypes (global cortical thickness [GCT] and global cortical surface area [GCSA] in 33 709 individuals and left-right subcortical volumes in 19 629 individuals) and alcohol use behaviors (alcoholic drinks per week [DPW] in 537 349 individuals, binge drinking frequency in 143 685 individuals, and alcohol use disorder in 8845 individuals vs 20 657 control individuals [total of 29 502]).

**Results:**

The main bidirectional MR analyses were performed in samples totaling 763 874 individuals, among whom more than 94% were of European ancestry, 52% to 54% were female, and the mean cohort ages were 40 to 63 years. Negative associations were identified between genetically predicted GCT and binge drinking (β, −2.52; 95% CI, −4.13 to −0.91) and DPW (β, −0.88; 95% CI, −1.37 to −0.40) at a false discovery rate (FDR) of 0.05. These associations remained significant in multivariable MR models that accounted for neuropsychiatric phenotypes, substance use, trauma, and neurodegeneration. TWAS of GCT and alcohol use behaviors identified 5 genes at the 17q21.31 locus oppositely associated with GCT and binge drinking or DPW (FDR = 0.05). Cell-type enrichment analyses implicated glutamatergic cortical neurons in alcohol use behaviors.

**Conclusions and Relevance:**

The findings in this study show that the associations between GCT and alcohol use may reflect a predispositional influence of GCT and that 17q21.31 genes and glutamatergic cortical neurons may play a role in this association. While replication studies are needed, these findings should enhance the understanding of associations between brain structure and alcohol use.

## Key Points

**Question:**

Are there directional associations between cortical or subcortical macrostructure and alcohol use?

**Findings:**

This mendelian randomization study including 763 874 participants in UK Biobank, Enhancing NeuroImaging Genetics through Meta Analysis (ENIGMA), Psychiatric Genomics Consortium (PGC), and GWAS & Sequencing Consortium of Alcohol and Nicotine use (GSCAN) studies identified a significant negative association between genetically predicted global cortical thickness and alcohol consumption and binge drinking. Downstream multiomic analyses indicate that 17q21.31 genes and glutamatergic cortical neurons contribute to this association.

**Meaning:**

The results from this study support emerging literature suggesting that cortical structure is associated with alcohol use and identify transcriptomic and cellular associations between these phenotypes that warrant further investigation.

## Teaser

This mendelian randomization study evaluates associations between brain structure and alcohol use.

## Introduction

Alcohol misuse causes large health and economic burdens globally^1,2^ and is a leading risk factor for premature death and disability in individuals aged 15 to 49 years.^1^ Heavy alcohol consumption impairs the nervous system,^3,4,5,6^ which may lead to neurological, cognitive, and psychiatric health ramifications.^7^ Altered macroscale brain structure is associated with psychopathology^8^ and could represent a mechanistic link between alcohol-associated neurotoxicity and health outcomes. Studies have consistently associated greater alcohol use and alcohol misuse with lower cortical and subcortical volumes.^9,10,11,12,13,14,15,16^ However, the directionality of these associations remains unclear, with some studies suggesting a predispositional impact of brain anatomy on alcohol use,^9,11,13,17^ challenging the notion that brain structure changes as a result of alcohol exposure.^14,18,19,20,21^

Robert et al^13^ analyzed longitudinal adolescent brain imaging data among 726 individuals and concluded that a greater rate of gray matter atrophy in frontal and temporal regions may lead to greater frequency of drunkenness. Similarly, a 2021 latent causal variable analysis^11^ suggested that greater pars opercularis volume, greater cuneus thickness, and lower basal forebrain volume were associated with increased alcohol misuse. By contrast, other studies continue to suggest that alcohol use alters neuroanatomy. For example, a 2021 co-twin study^18^ among 436 individuals found that alcohol exposure and genetic predisposition to alcohol use decreased thickness in multiple cortical regions. These studies highlight the ongoing debate regarding the directionality of associations between brain structure and alcohol use. Randomized clinical trials conducted to infer causality cannot be ethically or practically applied to study these associations and tens of thousands of participants may be required to identify replicable associations between brain magnetic resonance imaging (MRI) measures and behavioral traits.^22^ Alternative approaches using large data sets are needed to characterize associations between brain structure and alcohol use.

Recently developed genomics methods, including latent causal variable analysis and mendelian randomization (MR), facilitate the identification of directional associations between genetically influenced variables from population-based observational data and have been underapplied to questions regarding alcohol use and brain structure.^18,23,24,25^ Latent causal variable analysis only evaluates 2 phenotypes^26^ and does not explicitly test bidirectional associations.^27^ By contrast, MR is frequently used to evaluate directionality in neuropsychiatry.^28^ The multivariable extension of MR (MVMR) enables the assessment of multiple exposures to identify the direct association of each exposure with an outcome,^29^ which could help clarify the associations between brain structure and alcohol consumption accounting for potential mediating or confounding phenotypes.

In this study, we investigated associations between brain anatomy and alcohol use using summary-level genome-wide association study (GWAS) data for brain MRI measures and alcohol-related phenotypes. Our primary MR associated genetically predicted global cortical thickness (GCT) with alcohol use. We investigated whether GCT broadly associates with substance use by evaluating its association with smoking. Given differences in alcohol use patterns between men and women,^30^ we examined sex-specific associations between GCT and alcohol use. Next, we used MVMR accounting for confounding or mediating phenotypes to test the robustness of our GCT findings and performed multiomic analyses, including transcriptomic imputation^31^ and cell-type enrichment analysis,^32^ to describe the biological underpinnings of GCT–alcohol use associations.

## Methods

**Figure 1.**
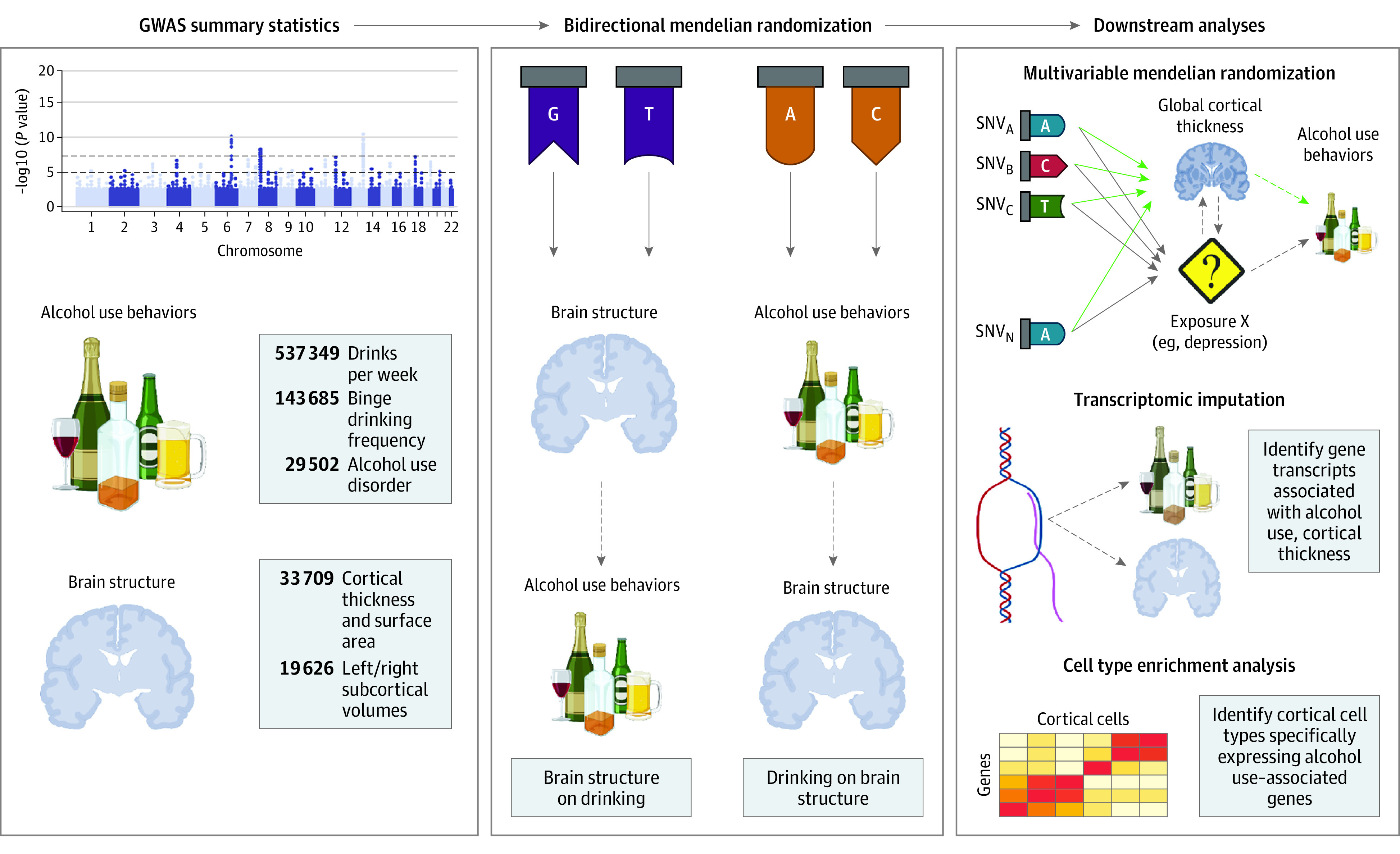
Study Overview GWAS indicates genome-wide association study; SNV, single-nucleotide variant.

Figure 1 presents a study overview. This study is reported in accordance with the Strengthening the Reporting of Observational Studies in Epidemiology (STROBE) reporting guideline (eTable 1 in Supplement 1).^33^ This study uses deidentified publicly available data, so no ethical approval from an institutional review board was required. The study protocol was not preregistered.

### Data Sources

Summary-level data were obtained from GWAS. Included GWAS have existing ethical permissions from their respective institutional review boards and include participant informed consent with rigorous quality control. Participants with missing phenotypic data were excluded from source GWAS. Exact data for sex and ethnicity were not available from all sources and are reported here as approximate percentages.

#### Cortical and Subcortical Structure

Our primary analyses evaluated 19 measures of cortical and subcortical brain structure sampled with MRI. We analyzed measures of global cortical thickness (GCT) and global cortical surface area (GCSA) from a recent GWAS of T1-weighted MR images from 1.5-3 T scans (n = 33 709).^34^ We performed secondary analyses on 34 regional cortical measures (eMethods in Supplement 1), but emphasized global averages rather than regional phenotypes because global measures may be less impacted by interindividual neuroanatomical variability,^35^ the limited functional relevance of gyral-based atlases like the Desikan-Killiany atlas used by Grasby et al,^34,36^ and the multiple-testing burden associated with a hypothesis-free regional analysis. GCSA was measured at the gray-white matter boundary and GCT was defined as the average distance between white matter and pial surfaces across both cortical hemispheres.^34^ Images were processed using FreeSurfer.^34,37^

Additionally, because a previous mega-analysis identified left-right hemispheric asymmetry in associations between subcortical structure and alcohol use,^12^ we focused our subcortical analysis on left-right volumes (n = 19 629).^38^ We evaluated 17 left-right volumes derived from T1-weighted MR images from 1.5-3 T scans: amygdala, hippocampus, accumbens, putamen, pallidum, thalamus, insula, caudate, and brainstem (combined volume). Mindboggle 101 atlases were used to label subcortical structures.^38^ MRI data were processed using advanced normalization tools.^38^ In exploratory bidirectional analyses, we analyzed phenotypes from a GWAS of overall subcortical volumes.^39^ We also investigated the association of genetically predicted alcohol consumption with longitudinal changes in brain structure (eMethods in Supplement 1).

#### Alcohol Use Behaviors

We used 3 GWAS of alcohol use behaviors in predominantly EA samples: a meta-analysis GWAS^40^ of alcoholic drinks consumed per week (DPW) (n = 537 349), a GWAS^41^ of binge drinking frequency among participants in the UK Biobank (n = 143 685), and a case-control GWAS^42^ of alcohol use disorder (AUD) (8845 individuals with AUD and 20 657 control individuals; total n = 29 502). Further description of these studies can be found in the eMethods in Supplement 1.

### MR

#### MR Instruments

The eMethods in Supplement 1 provides detailed methodology for instrument clumping, evaluations of instrument strength, sample overlap, procedures for missing instrument data, and testing of MR assumptions (eFigure 1 in Supplement 1). Our DPW and global cortical structure instruments included all genome-wide significant (GWS) single-nucleotide variants (SNVs) at a threshold of *P* < 5 × 10^−8^. Like previous neuropsychiatric MR studies evaluating exposures with few GWS SNVs,^43,44^ we used a *P* value threshold of 5 × 10^−6^ to select AUD, binge drinking, subcortical structure, and regional thickness instruments (eTable 3 in Supplement 1 and eTables 4-6 in Supplement 2). We performed MR using SNVs within the *ADH1B* (alcohol dehydrogenase 1B) locus, a primary enzyme in alcohol metabolism,^45^ as sensitivity analyses further assessing relationships of alcohol use on brain structure (eMethods in Supplement 1).

We evaluated our main GCT findings with additional MR. First, we tested the association of 34 regional cortical thickness exposures^34^ with alcohol use. Next, we evaluated the associations of GCT and smoking behaviors and examined potential sex-specific associations of GCT and alcohol use using sex-specific alcohol use GWAS data from the UK Biobank. Finally, we performed 11 MVMR analyses (eFigures 2 and 3 in Supplement 1) accounting for neuropsychiatric phenotypes, substance use, trauma, and neurodegeneration. We concatenated, extracted, and harmonized the independent instrument sets for GCT and the controlled-for exposure with each alcohol use behavior using standard MVMR methods (eMethods in Supplement 1 and eTables 7-17 in Supplement 2).^46^ We also performed leave-1-out MR^47^ and investigated the biological function of the GCT instrument with a gene-set enrichment analysis (eMethods in Supplement 1).

#### MR Statistical Analysis

We used the conventional inverse-variance weighted estimator (IVW) as our primary MR method. We supplemented IVW MR with MR-Egger, weighted median, weighted mode, and simple mode estimators, which rely on different assumptions than IVW.^48,49,50^ Evaluating multiple estimators facilitates the assessment of the robustness of MR estimates and is important in MR studies evaluating complex traits (ie, GCT) where the biological function of the instruments is unknown.^51^ Additionally, we used the Cochran Q heterogeneity test to evaluate heterogeneity in instrument effects, as heterogeneity may indicate violations of IVW assumptions.^52^ We used MR-PRESSO^53^ and MR-Lasso^54^ to obtain MR estimates with heterogenous SNVs removed. Finally, we used the MR Steiger directionality test for reverse causality in our bidirectional MR.^55^

We conservatively defined significance at a false discovery rate (FDR) of 0.05 for each MR analysis (eMethods in Supplement 1). We also discuss nominally significant results (*P* < .05). We report MR estimates as β values representing a change in outcome units per change in exposure unit. The unit for DPW was log-transformed,^40^ AUD was a binary measure, and binge drinking frequency was a categorical measure quantified as (0) never (1) less than monthly (2) monthly (3) weekly (4) daily/almost daily. Brain structure phenotypes were quantified as cortical thickness (mm); cortical surface area (mm^2^), and subcortical volumes (cm^3^) (eMethods in Supplement 1).

### Transcriptome-Wide Association Studies (TWAS)

We used the FUSION method^31^ to identify gene transcript-level associations with the alcohol use and brain structure phenotypes. To perform TWAS, we integrated alcohol use and GCT GWAS summary statistics with cortical RNA sequence reference panels from the CommonMind Consortium^56^ and the Genotype-Tissue Expression Consortium^57^ (eMethods in Supplement 1).

### Cell-Type Enrichment Analyses

We used Cell-Type Expression-Specific Integration for Complex Traits ^32^ with default parameters to perform cell-type enrichment analyses using the alcohol-associated GWAS data and single-cell RNA sequencing data of 120 cortical cell types (56 excitatory neurons, 54 inhibitory neurons, and 10 nonneuronal cells) from the Allen Brain Map Human Multiple Cortical Areas SMART-sequence data set^58^ (eMethods in Supplement 1).

## Results

### Bidirectional MR Reveals Negative Association Between Genetically Predicted Global Cortical Thickness and Alcohol Use Behaviors

**Figure 2.**
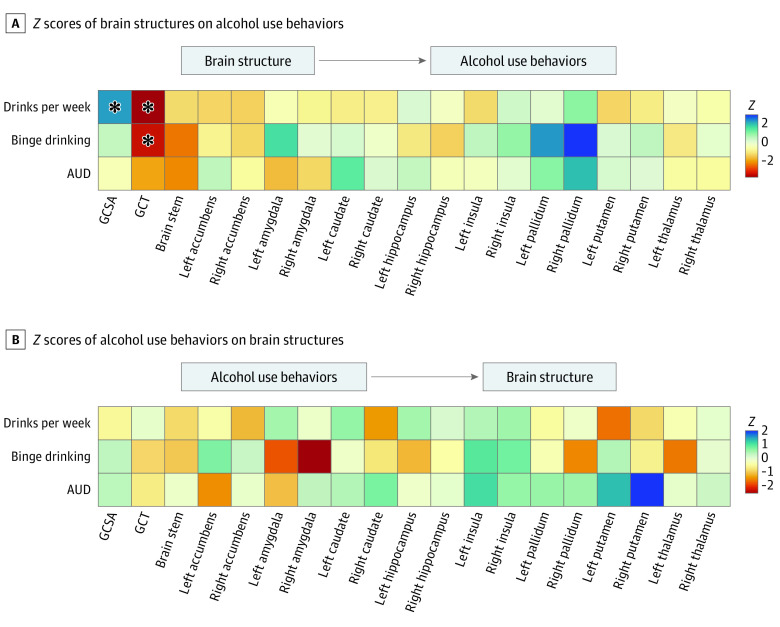
Bidirectional Mendelian Randomization of Brain Structures and Alcohol Use Behaviors Results from the bidirectional 2-sample mendelian randomization analyses of brain structures and alcohol use behaviors. All *Z* scores (β [SE]) were derived using inverse-variance weighted estimator (IVW) methodology. A, *Z* scores of the 19 brain structures included in the primary MR analyses on alcohol use behaviors. B, *Z* scores of alcohol use behaviors on the 19 brain structures. Asterisk indicates significance at a false discovery rate of 0.05. IVW results were prioritized in our interpretation of the results, while other methods served to assess the robustness of IVW estimates. See eTables 18-21 in Supplement 2 for full results.

The main bidirectional MR analyses included 763 874 individuals who were predominantly of European ancestry (more than 94%). Cohorts had mean ages between 40 and 63 years, and 52% to 54% of included individuals were female (eTable 2 in Supplement 1). Analyses revealed significant associations of GCT with alcohol use at FDR = 0.05. These associations were unidirectional. The MR analyses failed to find any nominally significant associations between genetically predicted alcohol use and GCT (eTables 18 and 19 in Supplement 2). Conversely, we found that genetically predicted GCT has a negative association with DPW and binge drinking frequency (DPW β, −0.88; 95% CI −1.36 to −0.40; *P* = 3.58 × 10^−4^; binge drinking β, −2.52, CI; −4.13 to −0.91; *P* = .002) (Figure 2; IVW estimators are presented unless otherwise specified). The associations between GCT, DPW, and binge drinking remained significant using weighted median and MR-Lasso estimators, supporting the validity of the IVW estimate (Table 1). Regarding associations between global cortical surface area (GCSA) and alcohol phenotypes, one finding suggested genetically predisposed GCSA was positively associated with DPW (β, 3.87 × 10^−6^; 95% CI, 1.16 × 10^−6^ to 6.59 × 10^−6^; *P* = .005); however, other MR methods did not corroborate this association. Additionally, unlike GCT, GCSA was not associated with binge drinking.

**Table 1.** Mendelian Randomization (MR) of Global Cortical Thickness (GCT), Alcoholic Drinks Per Week (DPW), and Binge Drinking Frequency Using Complementary MR Estimators

Exposure	Outcome	MR estimator	No. of instrument SNVs	Effect estimate, β (95% CI)	*P* value
GCT	DPW	IVW	5	−0.88 (−1.36 to −0.40)	3.58 × 10^−4^
		MR Egger	5	−2.26 (−4.12 to −0.40)	.098
		MR Lasso	5	−0.88 (−1.36 to −0.40)	3.58 × 10^−4^
		Weighted median	5	−0.80 (−1.33 to −0.28)	.003
		Simple mode	5	−0.77 (−1.51 to −0.03)	.11
		Weighted mode	5	−0.78 (−1.54 to −0.03)	.11
	Binge drinking frequency	IVW	6	−2.52 (−4.13 to −0.91)	.002
		Post-PRESSO IVW	5	−1.64 (−2.66 to −0.63)	.002
		MR Egger	6	−6.91 (−14.01 to 0.19)	.13
		MR Lasso	5	−1.64 (−2.66 to −0.63)	.002
		Weighted median	6	−2.13 (−3.36 to −0.90)	6.75 × 10^−4^
		Simple mode	6	−1.83 (−3.82 to 0.17)	.13
		Weighted mode	6	−1.57 (−3.31 to 0.17)	.14

Abbreviations: IVW, inverse-variance weighted estimator; SNV, single-nucleotide variant.

Our tests of left-right subcortical volumes on alcohol behaviors yielded 3 significant results using secondary MR methods (FDR = 0.05) (eTable 20 in Supplement 2). Greater right pallidum volume was unidirectionally associated with increased binge drinking (IVW-PRESSO β, 0.063; 95% CI, 0.023 to 0.10; *P* = .002; LASSO β, 0.06; 95% CI, 0.03 to 0.09; *P* = 3.69 × 10^−4^) and greater risk for AUD (LASSO β, 0.25; 95% CI, 0.09 to 0.40; *P* = .002) (Figure 2). Genetically predicted right pallidum volume was nominally associated with binge drinking and AUD (binge β, 0.08; 95% CI, 0.030 to 0.13; *P* = .001; AUD β, 0.18; 95% CI, 0.017 to 0.35; *P* = .03). The other nominally significant unidirectional associations between subcortical volumes and alcohol use were left pallidum with binge drinking (β, 0.06; 95% CI, 0.01 to 0.10; *P* = .01), brain stem with binge drinking (β, −0.04, 95% CI, −0.07 to −0.00; *P* = .03), and brain stem with AUD (β, −0.17; 95% CI, −0.33 to −.00; *P* = .047). The right pallidum was the subcortical structure most strongly associated with alcohol use in this study. The genetically predicted volume of this region was positively associated with alcohol use across methodologies and alcohol use behaviors. Beyond the right pallidum, we failed to robustly connect subcortical volumes with alcohol use. Additionally, unlike our GCT finding, our pallidum finding was not corroborated by weighted median analysis or a significant association with DPW.

MR identified nominally significant unidirectional associations between binge drinking and right amygdala volume (β, −0.19; 95% CI, −0.35 to −0.04; *P* = .01) and between AUD and right putamen volume (β, −0.04; 95% CI, 0.00 to 0.08; *P* = .04) (Figure 2; eTable 21 in Supplement 2). However, exploratory bidirectional results using overall subcortical volumes were null (eTables 22 and 23 in Supplement 2). MR estimates using cis-*ADH1B* instruments were also null for both cortical and subcortical structures (eTables 24-26 in Supplement 2), as were exploratory estimates of the associations of alcohol use with age-independent and age-dependent longitudinal changes in brain structure (eTables 27-30 in Supplement 2). Ultimately, our most robust finding was an association between genetically predicted GCT and alcohol use, motivating the focus of our downstream analyses.

### MR Testing the Robustness of GCT-Alcohol Consumption Associations

To investigate whether specific cortical regions underlie the association between GCT and alcohol use, we performed MR using regional thickness phenotypes as exposures and DPW, binge drinking, and AUD as outcomes. No results approached FDR significance (eResults in Supplement 1 and eTables 31, 32, and 33 in Supplement 2). Additionally, we failed to find an association between genetically predicted GCT and smoking, and exploratory MR identified no sex differences in GCT-alcohol consumption associations (eResults in Supplement 1 and eTables 34 and 35 in Supplement 2). Leave-1-out analyses found no evidence of high influence variants among GCT instrument SNVs (eFigure 4 and eTable 36 in Supplement 1). In 11 MVMR models jointly assessing GCT and possible confounding exposures on alcohol use, GCT retained robust, statistically significant associations with DPW and binge drinking (eMethods in Supplement 1 and eTable 37 in Supplement 2). The GCT MVMR estimates were similar in magnitude and direction to the corresponding single-variable estimates except in the MVMR model accounting for cognition. In this model, the GCT estimate on DPW was reduced by 29.5% (from β, −0.88 to β, −0.62) relative to the single-variable estimate, while the estimate on binge drinking was reduced by 46.4% (from β,−2.52 to β, −1.35). MVMR-IVW estimates were broadly consistent with MVMR-Egger sensitivity analyses; however, the MVMR-Egger estimates were substantially less precise. MVMR-Egger intercept analysis did not indicate horizontal pleiotropy (eTable 37 in Supplement 2).

### Genes at the 17q21.31 Locus Were Oppositely Associated With GCT and Alcohol Use

We performed TWAS on GCT and alcohol consumption using 3 cortical transcriptomic expression and splicing reference panels^56,57^ (Table 2). eTables 38-41 in Supplement 2 contain the full TWAS results. We identified 8 protein-coding genes associated with both GCT and 1 or more alcohol use behaviors: *ACTR1B*, *PLEKHM1*, *LRRC37A2*, *CRHR1*, *ARHGAP27*, *WNT3*, *RTN1*, and *LRRC37A*. Five of these genes, *PLEKHM1*, *LRRC37A2*, *CRHR1*, *ARHGAP27*, and *LRRC37A*, were oppositely associated with GCT and alcohol consumption (eTable 42 in Supplement 1). All 5 of these genes are contained within the 17q21.13 locus.

**Table 2.** FUSION^31^ Transcriptome-Wide Association Studies (TWAS): Protein Coding Genes Oppositely Associated With Global Cortical Thickness (GCT) and Alcohol Consumption

Gene	Locus	*Z* score: thickness	Reference panel: thickness	Significant alcohol use associations	*Z* score(s): alcohol use	Top reference panel(s): alcohol use
*PLEKHM1*	17q21.31	5.59	GTEx cortex	Binge drinking, DPW	−5.77, −5.34	GTEx cortex, GTEx cortex
*CRHR1*	17q21.31	−5.16	CMC DLPFC: splicing	Binge drinking, DPW	5.09, 4.75	CMC DLPFC: splicing, CMC DLPFC: splicing
*ARHGAP27*	17q21.31	4.89	CMC DLPFC	Binge drinking, DPW	−4.87, −4.50	CMC DLPFC, CMC DLPFC
*LRRC37A2*	17q21.31	5.30	GTEx cortex	Binge drinking	−6.13	GTEx cortex
*LRRC37A*	17q21.31	3.75	GTEx cortex	Binge drinking	−4.21	GTEx cortex
*ACTR1B*	2q11.2	6.11	CMC DLPFC: splicing	DPW	3.06	CMC DLPFC: splicing
*WNT3*	17q21.31	4.62	GTEx cortex	DPW	3.11	GTEx cortex
*RTN1*	14q23.1	4.15	CMC DLPFC: splicing	DPW	2.97	CMC DLPFC: splicing

Abbreviations: CMC, CommonMind Consortium; DLPFC, dorsolateral prefrontal cortex; DPW, alcoholic drinks per week; GTEx, Genotype-Tissue Expression Consortium.

### Cell-Type Enrichment Analysis–Associated Glutamatergic Cortical Neurons With Alcohol Consumption

**Figure 3.**
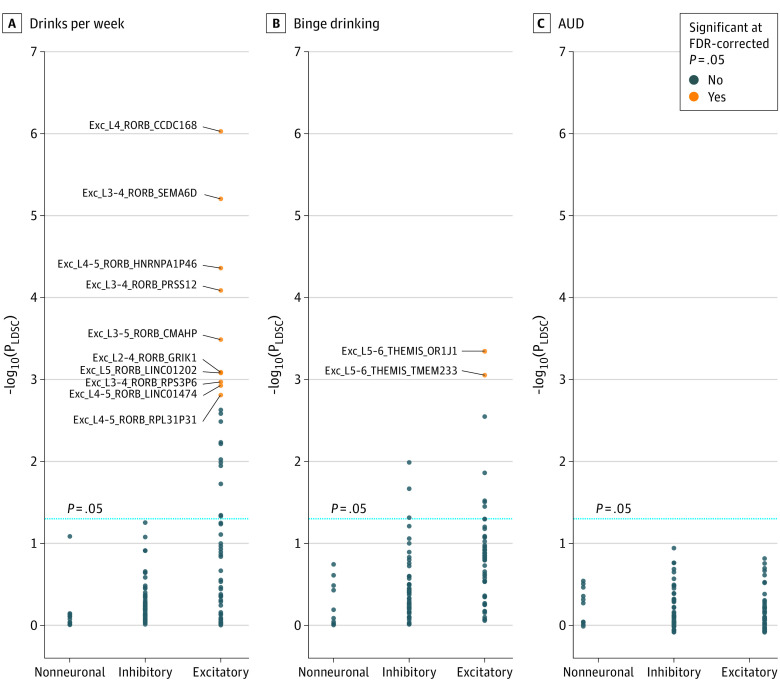
Cell-Type Expression-Specific Integration for Complex Traits–Cell-Type Enrichment Analyses A total of 120 cortical cell types were analyzed from the Allen Brain Map Human Multiple Cortical Areas SMART-sequence data set. Cell types are organized by broad class: excitatory (56 cell types), inhibitory (54 cell types), and nonneuronal (10 cell types). Thirty distinct cell types were nominally significant (*P* < .05); 27 were excitatory, and 3 were inhibitory. See eTable 43 in Supplement 1 for full results and eMethods in Supplement 1 for a full explanation of cell type nomenclature. AUD indicates alcohol use disorder; LDSC, linkage disequilibrium score regression.

eTable 43 in Supplement 2 contains full results from our cell-type enrichment analysis. We found a total of 31 nominally significant associations between a cell type and a alcohol use behavior representing 30 distinct cell types, 27 of which are excitatory glutamatergic cells and 3 of which are inhibitory GABAergic cells. Twelve excitatory cell types remained significant at FDR = 0.05, including 10 cells associated with DPW and 2 associated with binge drinking (Figure 3).

## Discussion

This MR study used large population-based data on the genetic architecture of cortical and subcortical structure,^34,38^ MR, and novel multiomic methods to identify directional and biological associations between human brain structure and alcohol use. Our large sample sizes (between 19 629 and 537 349 participants^34,38,40,41,42^) increased statistical power relative to previous brain structure–alcohol consumption studies.^13,18^ Our findings suggest that a predisposition toward lower GCT may be associated with greater alcohol consumption and binge drinking. Conversely, we failed to find strong evidence that a genetic predisposition for alcohol use was associated with brain structure or its longitudinal plasticity.

More modestly, our study suggests genetically predicted right pallidum volume was positively associated with alcohol consumption. This finding was not replicated in either our MR of overall subcortical volumes or a recent MR by Logtenberg et al^59^ investigating substance use and overall subcortical volumes. Additionally, while Logtenberg et al^59^ associated alcohol dependence with reduced overall amygdala and hippocampal volumes, after multiple testing corrections, we failed to associate a genetic liability for binge drinking, DPW, AUD, or an *ADH1B* instrument with these regions using hemispheric, overall, and longitudinal subcortical outcomes. Discrepancies between our studies may have resulted from differences in statistical methodology, power, or the specific phenotypes evaluated (eDiscussion in Supplement 1).

Our consistent identification of an association between genetically predicted GCT and alcohol use behaviors across MR methods and sex-specific analyses implicates the cortex as a potential driver of vulnerability to alcohol consumption and binge drinking. Interestingly, GCT had no association with smoking, suggesting its association with alcohol consumption may not reflect a broader association with substance use. Our failure to identify specific cortical regions associated with alcohol use may mean larger data sets are needed to characterize such associations. Importantly, GCT estimates from MVMR analyses remained significant when accounting for 11 potential mediators or confounders. The reduction in GCT effect estimates in MVMR models accounting for cognition suggests mediation of the GCT-alcohol use associations, especially given the significant MVMR estimates for cognition on alcohol use (eTable 37 in Supplement 2). Additionally, while we failed to find evidence for alcohol-associated cortical thinning in a population of adults with a mean (SD) age of 40 (8) years, alcohol use could cause cortical thinning in younger adults and adolescents due to increased cortical plasticity during these developmental stages.^19^ For instance, recent work analyzing young adults showed that alcohol use predisposition leads to decreased thickness of cortical control and salience networks.^18^ While participant age and other methodological particularities may influence the results of studies investigating alcohol–brain structure interactions, we found that for a middle-aged population, alcohol use primarily followed cortical anatomy.

Our investigation of the transcriptomic relationship between GCT and alcohol use identified 5 protein coding genes oppositely associated with GCT and alcohol use behavior: *PLEKHM1*, *LRRC37A2*, *CRHR1*, *ARHGAP27*, and *LRRC37A*. These 5 genes could contribute to the negative association between GCT and alcohol use. All 5 are located at 17q21.31. This locus, characterized by extensive linkage disequilibrium,^60^ is the site of 2 haplotypes: the inverted H2 haplotype (found in approximately 20% of individuals of European ancestry), and the H1 haplotype.^61^ Comparing our imputed transcriptomes with limited cortical RNA-sequence data and past association studies suggests that lower GCT and greater alcohol use may be associated with the H1 haplotype.^62^^,^ Notably, *CRHR1* encodes a G-protein coupled receptor that binds corticotropin-releasing hormone and its agonists. In line with our findings, *CRHR1* upregulation in the amygdala^63,64^ and cortex^63^ have been associated with greater alcohol consumption and dependence. Additionally, previous studies have suggested that *CRHR1* modulates the behavioral and cognitive outcomes associated with stress.^65,66^ *CRHR1* may also affect cortical macrostructure, as previous studies indicate *CRHR1* overexpression may be associated with early life stress-induced neuroanatomical changes and dendritic spine loss,^67,68^ suggesting a potential mechanism whereby early life stress interacts with *CRHR1* to impact cortical structure, leading to behavioral adaptations and harmful alcohol use. We present this hypothesis cautiously due to *CRHR1*’s location in a linkage disequilibrium block containing genes like *MAPT*, which may be involved in neurodegenerative diseases and cortical anatomy.^69^

Our cell-type analysis also found that excitatory neurons may underlie GCT’s association with alcohol use. These data support the notion that glutamatergic transmission plays an important role in alcohol misuse.^70,71^ Interestingly, *CRHR1* is expressed in glutamatergic, but not GABAergic, cortical neurons.^72^ Activation of *CRHR1* in the forebrain is associated with alteration in glutamatergic neurotransmission and increased behavioral susceptibility to stress in mice.^72^ Therefore, our single-cell findings support our hypothesis associating cortical *CRHR1* expression with increased stress susceptibility, cortical thinning, and alcohol misuse.

### Limitations

This study has several limitations. First, MR instrumentation in neuropsychiatry remains challenging due to the complexity of the phenotypes and frequent uncertainty of genetic variants’ biological functions.^28^ For several of our alcohol use phenotypes, we used a relaxed *P* value threshold due to the limited number of variants at GWS, in line with previous psychiatric MR studies.^43,44^ While these relaxed thresholds could introduce weak instrument bias or increase the possibility of horizontal pleiotropy, all instrument SNVs had *F* statistics exceeding 10, the conventional cutoff for designating strong instruments.^73^ To protect our MR estimates from the influence of invalid instruments and violations of MR’s core assumptions, we used sensitivity analyses (eg, Steiger directionality test, *ADH1B* instrument, and leave-1-out), MR estimators with relaxed assumptions (eg, weighted median and post-Lasso IVW), and MVMR accounting for possible confounding or mediating traits, which yielded largely consistent results and suggested minimal violations of MR’s assumptions.^51^ However, causal inference requires triangulating evidence,^74^ and we emphasize that our results should be interpreted in the context of other studies investigating similar questions with different methodologies.^9,11,13,17^ Furthermore, we recognize the neuroanatomical phenotypes we analyzed may not fully encapsulate brain damage and caution that our null findings do not imply that alcohol does not affect brain health.

## Conclusions

The results of this study provide evidence that genetically predicted GCT was associated with alcoholic drinks consumed per week and binge drinking frequency after accounting for neuropsychiatric phenotypes, substance use, trauma, and neurodegeneration. We also found that several genes located at 17q21.31 and glutamatergic cortical neurons may be biological mechanisms associating GCT with alcohol consumption. These findings should be replicated in larger samples to better understand the interactions between brain structure and alcohol use.

## Notes

Supplement 1. eMethods eResults eDiscussion eReferences eFigure 1. Mendelian randomization model and assumptions eFigure 2. Multivariable Mendelian randomization: methodological overview eFigure 3. Possible pleiotropic models explaining relationships between SNP instruments, global cortical thickness, and alcohol use behaviors eFigure 4. Results of leave-one-out analyses of GCT on drinks per week and binge drinking eTable 1. STROBE-MR reporting guidelines eTable 2. Demographics of GWAS cohorts featured in single-variable MR analyses eTable 3. ENIGMA global cortical thickness and surface area instrument SNPs eTable 36: Leave-one-out MR of GCT on DPW, binge drinking frequency eTable 42: Unique TWAS-identified genes significant (FDR=0.05) for both cortical thickness, alcohol use behavior(s)

Supplement 2. eTable 4. Alcohol use behavior instrument SNPs eTable 5. Subcortical volume instrument SNPs eTable 6. Regional cortical thickness instrument SNPs eTable 7. Harmonized data for MV exposures: global cortical thickness and cognitive performance on alcohol use behavior outcomes eTable 8. Harmonized data for MV exposures: global cortical thickness and major depression on alcohol use behavior outcomes eTable 9. Harmonized data for MV exposures: global cortical thickness and ADHD on alcohol use behavior outcomes eTable 10. Harmonized data for MV exposures: global cortical thickness and schizophrenia on alcohol use behavior outcomes eTable 11. Harmonized data for MV exposures: global cortical thickness and bipolar disorder on alcohol use behavior outcomes eTable 12. Harmonized data for MV exposures: global cortical thickness and post-traumatic stress disorder on alcohol use behavior outcomes eTable 13. Harmonized data for MV exposures: global cortical thickness and life-time trauma on alcohol use behavior outcomes eTable 14. Harmonized data for MV exposures: global cortical thickness and sleep disorders on alcohol use behavior outcomes eTable 15. Harmonized data for MV exposures: global cortical thickness and Alzheimer's disease on alcohol use behavior outcomes eTable 16. Harmonized data for MV exposures: global cortical thickness and cannabis use on alcohol use behavior outcomes eTable 17. Harmonized data for MV exposures: global cortical thickness and packyears adult smoking on alcohol use behavior outcomes eTable 18. MR of global cortical thickness, surface area on alcohol use behaviors eTable 19. MR of alcohol use behaviors on global cortical thickness, surface area eTable 20. MR left/right subcortical volumes on alcohol use behaviors eTable 21. MR of alcohol use behaviors on left/right subcortical volumes eTable 22. MR analysis of combined hemispheric subcortical volume on alcohol consumption eTable 23. MR analysis of alcohol use behavior exposures on combined hemisphere subcortical volumes eTable 24. MR analysis of cis-instruments for ADH1B on ENIGMA full and regional area surface area and thickness eTable 25. MR analysis of cis-instruments for ADH1B on left/right subcortical volumes eTable 26. MR analysis of cis-instruments for ADH1B on combined hemisphere subcortical brain volumes eTable 27. MR analysis of effects of alcohol use behaviors on change rate of brain structures (assuming age-independent change rate throughout lifespan) eTable 28. MR analysis of effects of alcohol use behaviors on change rate of brain structures (assuming age-dependent change rate throughout lifespan) eTable 29. MR analysis of cis instruments for ADH1B on age-independent change rates of cortical and subcortical brain structures eTable 30. MR analysis of cis instruments for ADH1B on age-dependent change rates of cortical and subcortical brain structures eTable 31. MR of regional cortical thickness on drinks per week (DPW) eTable 32. MR of regional cortical thickness on binge drinking eTable 33. MR regional cortical thickness on alcohol use disorder (AUD) eTable 34. MR of global cortical thickness on smoking behaviors eTable 35. Sex-specific results from MR of global cortical thickness on binge drinking frequency and amount of alcohol drunk on typical drinking day eTable 37. MVMR results for mv exposures global cortical thickness and cognitive performance, neuropsychiatric disorders and substance use and/or dependence on alcohol related behavior outcomes eTable 38. TWAS of global cortical thickness in 3 cortical reference panels eTable 39. TWAS of DPW in 3 cortical reference panels eTable 40. TWAS of binge drinking frequency in 3 cortical reference panels eTable 41. TWAS of AUD in 3 cortical reference panels eTable 43. CELLECT s-LDSC cell-type enrichment analysis, alcohol-related SNPs in human cortical reference panel
