# Supplementary material for: Association Between Brain Structure and Alcohol Use Behaviors in Adults: A Mendelian Randomization and Multiomics Study
Source: JAMA Psychiatry. 2022 Aug 10;79(9):869–78. doi: 10.1001/jamapsychiatry.2022.2196 (PMC9366661; doi:10.1001/jamapsychiatry.2022.2196)
Supplement: Supplement 1. — eMethods eResults eDiscussion eReferences eFigure 1. Mendelian randomization model and assumptions eFigure 2. Multivariable Mendelian randomization: methodological overview eFigure 3. Possible pleiotropic models explaining relationships between SNP instruments, global cortical thickness, and alcohol use behaviors eFigure 4. Results of leave-one-out analyses of GCT on drinks per week and binge drinking eTable 1. STROBE-MR reporting guidelines eTable 2. Demographics of GWAS cohorts featured in single-variable MR analyses eTable 3. ENIGMA global cortical thickness and surface area instrument SNPs eTable 36: Leave-one-out MR of GCT on DPW, binge drinking frequency eTable 42: Unique TWAS-identified genes significant (FDR=0.05) for both cortical thickness, alcohol use behavior(s) [file jamapsychiatry-e222196-s001.pdf]

Supplemental Online Content

Mavromatis LA, Rosoff DB, Cupertino RB, Garavan H, Mackey S, Lohoff FW. Association between brain structure and alcohol use behaviors in adults: a mendelian randomization and multiomics study. *JAMA Psychiatry*. Published online August 10, 2022. doi:10.1001/jamapsychiatry.2022.2196

eMethods

eResults

eDiscussion

eReferences

eFigure 1. Mendelian randomization model and assumptions

eFigure 2. Multivariable Mendelian randomization: methodological overview

eFigure 3. Possible pleiotropic models explaining relationships between SNP instruments, global cortical thickness, and alcohol use behaviors

eFigure 4. Results of leave-one-out analyses of GCT on drinks per week and binge drinking

eTable 1. STROBE-MR reporting guidelines

eTable 2. Demographics of GWAS cohorts featured in single-variable MR analyses

eTable 3. ENIGMA global cortical thickness and surface area instrument SNPs

eTable 36. Leave-one-out MR of GCT on DPW, binge drinking frequency

eTable 42. Unique TWAS-identified genes significant (FDR=0.05) for both cortical thickness, alcohol use behavior(s)

This supplemental material has been provided by the authors to give readers additional information about their work.

© 2022 Mavromatis LA et al. *JAMA Psychiatry.*

eMethods

Additional information about data sources:

Binge drinking: We assessed binge drinking through question 3 from the Alcohol Use Disorder Inventory Test (AUDIT) ("How often do you have six or more drinks on one occasion?"). We obtained binge drinking summary statistics from the Neale Lab GWAS (N=143 658 individuals of predominantly European ancestry).[1]

Alcohol use disorder: For alcohol use disorder (AUD), we used summary statistics from the PGC meta-analysis GWAS (N=8 845 cases and 20 657 controls of predominantly EA).[2] This GWAS measured alcohol dependence diagnoses, which were derived from clinician ratings or semi-structured interviews in which patients needed to meet at least three of the seven Diagnostic and Statistical Manual of Mental Disorders IV (DSM-IV)[3] criteria for alcohol dependence. Because of overlapping diagnostic criteria, a DSM-IV alcohol dependence diagnosis necessarily corresponds to a DSM-V AUD diagnosis.[3,4] Therefore, to reflect contemporary terminology, we refer to AUD, rather than alcohol dependence, throughout this study.

ENIGMA subcortical volumes: We used left/right subcortical volumes in our primary analysis to capture possible hemispheric asymmetries in the relationships between subcortical structures and alcohol use that have been reported previously.[5] We supplemented these analyses with a GWAS dataset from a recent Enhancing NeuroImaging Genetics through Meta-Analysis (ENIGMA) consortium meta-analysis that averaged left/right subcortical volumes into an overall measure for each structure.[6] For these analyses, we used the unrestricted GWAS summary statistics (N≤30 175 participants of European ancestry, average cohort age 10.0-83.8, cohort percentage female 0%-73%), from Satizabal et al. of 7 subcortical volumes (amygdala, caudate, putamen, pallidum, brainstem, thalamus, and nucleus accumbens).[6] T1-weighted images were processed using FreeSurfer, SPM, FSL-FIRST, MNI, in-house imaging software, and MIPAV. The left and right volumes were first calculated and then averaged to calculate the mean subcortical volumes for each structure (in cm^3).[6]

ENIGMA longitudinal changes in cortical and subcortical structures: Because 1) problematic alcohol use may impact the developing brain over time,[7] and 2) it has recently been shown that the genetic architecture of age-independent and age-dependent brain changes are distinct from the genetics of brain structure,[8] we performed additional MR analyses investigating the impact of alcohol use on longitudinal changes in brain structure. We used summary statistics from a recent ENIGMA meta-analysis GWAS of 40 cohorts on the genetic architecture of age-dependent and age-independent temporal changes in cortical and subcortical structure (15 640 participants of European ancestry).[8] 7 684 participants were female (49.1%) and the average age at baseline across the cohorts ranged from 7.3 to 77.3 years old across the cohorts (age range 4-99 years).[8] Cohorts were required to have longitudinal magnetic resonance imaging (MRI) and genotyping data.

This study featured T1-weighted MRI images from 1.5-3 Tesla scans that were processed using FreeSurfer.[8] Annual rates of change were computed for each participant by subtracting the baseline measurement for each of the 16 brain structure endpoints analyzed from the follow-up measurement and dividing by the duration of the follow-up.[8] Three separate GWAS analyses were performed to assess the age-independent and age-dependent impact of variants on 16 brain structure endpoints.[8] The meta-analysis performed to investigate the age-independent impact of variants used the model ($SNP_C \sim b_0 + \varepsilon_c$). The study used both a linear model ($Effect\_SNP_C \sim b_0 + b_1 \times age_c + \varepsilon_c$) and a quadratic model ($Effect\_SNP_C \sim b_0 + b_1 \times age_c + b_2 \times age_c^2 + \varepsilon_c$) to assess the age-dependent associations of SNPs with longitudinal changes in brain stuctures.[8]

To broadly align with the brain structures investigated in the primary bidirectional MR, we included endpoints related to longitudinal changes in average global cortical thickness, average cortical surface area, and seven subcortical structures (thalamus, caudate, putamen, pallidum, hippocampus, amygdala, and nucleus accumbens). We analyzed the three models (age-independent, linear age-dependent, and quadratic age-dependent) relating to each brain structure endpoint.

Mendelian randomization assumptions:

© 2022 Mavromatis LA et al. *JAMA Psychiatry.*

Mendelian randomization (MR) uses single nucleotide polymorphisms (SNPs) as instrumental variables to identify associations between the genetic liability for an exposure trait and an outcome.[9-11] The main assumptions underlying MR are 1) SNP instruments associate with the exposure trait of interest ("relevance assumption"), 2) the frequency of SNP instruments is not influenced by factors that also impact the outcome trait ("independence assumption"), and 3) SNP instruments only influence the outcome trait through the exposure trait ("exclusion restriction assumption").[12] (**eFigure1**). In the following sections, we describe the MR methods used in this study and how they are employed to test these assumptions.

Additional MR assessing the findings of primary bidirectional MR analyses:

After our initial bidirectional MR results showed that genetically-predicted GCT associated with binge drinking frequency and alcoholic drinks consumed per week, we performed additional MR analyses to test the robustness of these finding. We also used several of these analyses to test the MR independence and exclusion restriction assumptions (e.g., using multivariable MR (MVMR) to assess the relationships between GCT and alcohol use behaviors in the presence of neuropsychiatric, substance use, trauma-related, and neurodegenerative phenotypes that may confound these relationships).

*MR evaluating the association of variants within the ADH1B locus with brain structure***:** For our MR assessing the impact of alcohol use on brain structure, we tested the MR exclusion restriction assumption by using an additional alcohol use-related instrument comprised of SNPs located within or near the locus of *ADH1B* (alcohol dehydrogenase 1B), the primary enzyme responsible for the oxidation of alcohol to acetaldehyde (**eTables 24-26, 29, 30**).[13] Previous MR analyses have assessed the impact of alcohol use behaviors using *ADH1B* instruments on a wide-range of outcomes, including depression,[14] liver function,[15] cardiovascular disease,[16] cancer,[17] and many other traits.[17] Given these variants' proximities to the *ADH1B* gene and the well-known impact of *ADH1B* on alcohol metabolism, these variants are likely to have a relatively direct and specific impact on drinking. This analysis is important given the challenges of instrumentation in neuropsychiatric settings in which the exposures and outcomes are complex traits and the exact biological mechanisms through which variants impact exposures are often unknown.[18,19]

MR evaluating the association between genetically-proxied global cortical thickness and smoking: Alcohol use and tobacco smoking are likely to co-occur.[20,21] Alcohol use behaviors and smoking behaviors are also genetically linked.[22,23] Therefore, we investigated the association of genetically-proxied cortical thickness and several smoking behaviors to compare with our main cortical thickness-alcohol use results. We used four smoking-related endpoints from participants of European ancestry: cigarettes smoked per day (N=249 752),[20] pack years of smoking (N=142 387),[24] pack years of smoking as a proportion of lifespan exposed to smoking (N=142 387),[24] and light smoking (i.e., smoking at least 100 cigarettes during the participant's lifetime (N=123 894)).[24] We present the full results of this MR analysis in **eTable 34**.

MR evaluating the associations between genetically-proxied global cortical thickness and alcohol use in genetic males and females: We evaluated associations between genetically-proxied global cortical thickness and alcohol use behaviors in samples solely comprising males and other samples solely comprising females. To perform these analyses, we used the combined-sex SNP instruments for GCT contained in **eTable3** and sex-specific alcohol use datasets from the Neale Lab 2018 analysis of the UK Biobank.[1] We evaluated self-reported binge drinking frequency (N_{males}=48 855, $N_{females}$=59 630) and the average amount of alcohol consumed on a typical drinking session (N_{males}=48 784, $N_{females}$=59 472). The latter phenotype was defined as a response to the question "How many drinks containing alcohol of you have on a typical day when you are drinking?". An answer of 1-2 was coded as 1, 3-4 was coded as 2, 5-6 was coded as 3, 7-9 was coded as 4, and 10 or more was coded as 5. We evaluated the statistical significance of the differences in beta coefficients between males and females with a post-hoc hypothesis test. We report the full results of this analysis in **eTable 35** and further discuss our findings in **eResults**.

MVMR background and rationale: MVMR is an extension of two-sample MR that facilitates the simultaneous assessment of separate but correlated exposures (**eFigure 2**).[25,26] MVMR has been recently employed to disentangle the direct effect of each risk factor not mediated by other correlated risk factors for a range of health outcomes. For example, a recent study used MVMR to compare the causal role of substance use behaviors on the risk for COVID-19;[27] another used MVMR to evaluate the contributions of lipid subfractions on the risk for coronary artery

© 2022 Mavromatis LA et al. *JAMA Psychiatry.*

disease;[28] and another analyzed the relationships between neuropsychiatric disorders and autoimmunity.[29]

MVMR incorporates genetic variants from each risk factor into the same model, creating a single MVMR genetic instrument.[30] This permits estimation of the direct effect of each risk factor on the outcome of interest, excluding effects that result from common pleiotropic pathways and protecting effect estimates from confounding (**eFigure 3**). For example, consider our single-variable MR finding associating genetically-predicted global cortical thickness (GCT) with alcohol use behaviors. This relationship may be impacted by other neuropsychiatric disorders and behaviors that influence global cortical thickness and/or alcohol use. Vertical pleiotropy would be present if the SNPs instrumenting GCT impact alcohol use behaviors first via their impact on neuropsychiatric disorders, which, in turn, impact alcohol use behaviors (**eFigure 3A**). Similarly, horizontal pleiotropy could be present if SNP instruments for GCT impact alcohol use behaviors through an independent effect on neuropsychiatric disorders, which would violate the exclusion restriction assumption (**eFigure 3B**).[18] Finally, GCT SNP instruments may be subject to confounding bias if they impact the genetic propensity for neuropsychiatric endpoints but do not impact alcohol use behaviors (**eFigure 3C**).

MVMR is well-suited to evaluate the impact of neuropsychiatric mediators and confounders on the relationships between global cortical thickness and alcohol use behaviors and to generate MR estimates that are unconfounded/unmediated by the controlled for phenotype. While MVMR models may be extended to perform mediation analyses, which seek to explain and quantify potential pathways through which an exposure of interest impacts an outcome (i.e., quantification of the total effect, direct effect, and indirect effect),[31,32] we employed our MVMR analysis to test the robustness of the relationships between GCT and alcohol use behaviors like other recent studies.[29]

MVMR phenotypes: We constructed MVMR models by combining the GCT GWAS data with summary-level GWAS data from 11 phenotypes related to neuropsychiatric disorders, substance use, neurodegeneration, trauma, and cognition (derived from participants of European ancestry). Each MVMR model was comprised of variants instrumenting GCT and variants instrumenting one other endpoint.

We obtained schizophrenia (SCZ) summary statistics from the PGC meta-analysis GWAS of 49 cohorts (N=77 096 individuals of predominantly European ancestry).[33] Diagnoses were established based on clinical evaluation, semi-structured interviews, and medical records based on DSM-IV or ICD-10 criteria.[33]

We obtained bipolar disorder (BD) summary statistics from the PCG meta-analysis GWAS of 32 cohorts (N=20 352 cases and 31 358 controls of predominantly European ancestry).[34] BD is split into 2 clinical subtypes: bipolar I disorder (manic episodes that alternate with depressive episodes) and bipolar II disorder (at least one depressive and one hypomanic episode incident during a patient's lifetime).[34] Diagnoses were based on DSM-IV, ICD-9, or ICD-10 criteria from assessments done by trained interviewers, clinician-administered checklists, or medical record reviews.

We used a 2019 GWAS on a depression phenotype constructed by Howard et. al (70 756 cases and 329 443 controls, total N=500 199).[35] This GWAS meta-analyzed data on a broad depression phenotype from the UK Biobank along with data on major depressive disorder (MDD) from the Psychiatric Genetics Consortium (PGC). Cases of broad depression among participants in the UK Biobank were defined as either A) an answer of "Yes" to the question: "Have you ever seen a general practitioner/psychiatrist for nerves, anxiety, tension, or depression," or B) if participants met criteria for and were diagnosed with any of these depressive mood disorders as defined by the ICD codes: F32 – Single Episode Depression, F33 – Recurrent Depression, F34 – Persistent mood disorders, F38 – Other mood disorders, and F39 – Unspecified mood disorders. The PGC MDD GWAS was a meta-analysis that included GWAS of 7 cohorts of predominantly European ancestry. Patients were classified as having MDD through self-report, clinician interviews, and/or medical record diagnoses based on standard criteria.

We used ADHD data derived from the PGC GWAS comparing the genetics of clinical diagnoses of ADHD (20 183 cases and 35 151 controls, total N=55 374, European ancestry).[36]

We used Alzheimer's disease (AD) GWAS data from the Alzheimer's Disease Genetics Consortium 2019 meta-analysis (21 982 AD cases and 41 944 controls, total N=63 926, predominantly European ancestry).[37]

For cognitive performance, we used a 36-cohort 2018 meta-analysis GWAS on cognitive performance (N=257 841)[38] of participants of European ancestry. Cognitive performance was quantified as an individual's average

© 2022 Mavromatis LA et al. *JAMA Psychiatry.*

standardized score on a test of verbal-numerical reasoning taken at 4 timepoints and featuring 13 logic and reasoning questions. The test was designed to measure fluid intelligence.

For sleep-related conditions, we used GWAS data of multiple sleep disorders among predominantly European ancestry UK Biobank participants (N=261 194) from the MRC-IEU pipeline.[24]

Because our transcriptomic imputation associated *CRHR1* expression with both GCT and alcohol use behaviors, and because previous studies have shown that *CRHR1* overexpression impacts early life stress-induced neuroanatomical changes, including dendritic spine loss,[39,40] we included MVMR models incorporating stress- and trauma-related GWAS data. Specifically, we used the 2019 PGC PTSD meta-analysis (~30 000 cases and ~170 000 controls),[41] and the Coleman et al. 2020 GWAS (35 269 cases, 63 451 controls, N=98 720) assessing life-time exposure to traumatic events among participants in the UK Biobank. Traumatic events included 3 childhood events (did not feel loved, felt hated, sexually abused by a family member) and 4 adulthood events (age 18+, experienced physical violence, experienced belittlement, experienced sexual interference, experienced sexual assault). Cases included UK Biobank participants who reported experiencing 2 or more of these traumatic events.[42] UK Biobank participants reporting one traumatic event were excluded from the analysis. Controls were comprised of UK Biobank participants reporting 0 traumatic events.[42]

Finally, we included variables related to tobacco smoking and cannabis use to account for comorbid substance use. For tobacco smoking, we used the UK Biobank-derived GWAS of pack years of smoking as a proportion of life span exposed to smoking (N=142 387),[24] and the 2018 Pasman et al. GWAS on cannabis use (N=184 765).[43]

Two-sample MR: sample overlap:

Sample overlap between exposure and outcome datasets produce may bias effect estimates in two-sample MR.[44] We report the following sample overlap percentages for our primary two-sample MR analyses: Binge drinking frequency and cortical structure, up to 7.0%; binge drinking frequency and subcortical volumes, up to 13.7%. Simulation studies indicate that sample overlap bias is minimal when genetic instruments have large F-statistics[44] and when overlapping samples come from large biobanks,[45] suggesting that our results are minimally affected by this bias.

Two-sample and multivariable Mendelian randomization: R packages:

All MR and MVMR analyses were carried out using TwoSampleMR, version 0.5.5,[18] and MendelianRandomization, version 0.5.0,[46] in the R environment, version 4.0.2.

MR and MVMR procedures for instrument selection and clumping, evaluations of instrument strength, and missing instrument data:

For each exposure in our 11 MVMR models except for sleep disorders, instruments included all SNPs achieving genome-wide significance ($P<5\times10^{-8}$). For sleep disorders and our combined subcortical volume exposures, instruments were selected at $P<5\times10^{-6}$ due to a lack of genome-wide significant variants. All instrument SNPs were clumped at linkage disequilibrium (LD) r^2=.001 and a distance of 10 000 kb, using reference samples comprised of participants of European ancestry.[47]

We tested the relevance assumption (the first assumption of MR) by calculating the variance explained by the instrument (i.e., the R^2) and F-statistics for each instrument. By convention, F-statistics >10 provides evidence that the instruments are unlikely to be subject to weak instrument bias, which may occur when the variants comprising the MR instrument explain only a small proportion of the exposure resulting in reduced statistical power to reject the null hypothesis.[47] For our instruments, estimated F-statistics for the unconditional instruments generally exceeded 20, while 10 is the conventional threshold designating weak and strong MR SNP instruments,[47] suggesting minimal bias from weak instruments (**eTables**).[48]

Next, we extracted instrument SNPs from outcome datasets and harmonized effect alleles. Instrument SNPs that

were missing from outcome data were discarded prior to performing MR unless proxies were identified (LD r^2>.8). For MVMR, we were unable to calculate conditional F-statistics to assess the strength of the multivariable instrument sets; single-variable MR statistical methods recently extended to MVMR are only appropriate for non-overlapping exposure summary level data sources. When overlapping, the requisite pairwise covariances between SNP associations are only determinable using individual level data.[49] Harmonized MVMR datasets are presented in **eTables 7-17.**

For the MR analyses investigating the impact of alcohol consumption on brain structure using SNPs located within or near the *ADH1B* locus, we extracted SNPs within 100 kilobases of the *ADH1B* locus (chromosome 4:100 226 121-100 242 558 [GRCh37/hg19]) from the GWAS summary statistics for drinks per week performed by Liu et al.[50] We clumped using the procedure described above (LD r^2<.001, 10 000kb window), constructing two alternative instrument sets. The first consisted of two SNPs: rs1229984, base pair (bp)=100 239 319, $P<1\times10^{-200}$, F-statistic=927; and rs78234152, bp=100 279 889, $P=2.18\times10^{-19}$, F-statistic=81.1. The second, used when the outcome GWAS did not include rs1229984, consisted of three SNPs: rs141973904, bp=100 262 242, $P=2.51\times10^{-91}$, F-statistic=411; and rs283412, bp=100 267 672, $P=2.21\times10^{-13}$, F-statistic=53.8, in addition to rs78234152. F-statistics for each of these SNPs are much greater than the conventional F-statistic threshold of 10, indicating sufficient instrument strength for the *ADH1B* instrument.[44]

MR estimators:

<u>*MR:*</u> For the main bidirectional single-variable MR analysis investigating the total effects of each of the brain structure measure on alcohol use behaviors and vice versa, we used the conventional and statistically powerful inverse-variance weighted MR (IVW) as the primary method. The IVW estimator combines Wald ratio estimates for each instrument SNP in a fixed-effect meta-analysis in which SNP instruments are inversely weighted by the variance of the SNP-outcome association.[51] In the absence of unbalanced horizontal pleiotropy and assuming the instruments are valid, MR IVW returns unbiased estimates of a causal effect.[18,52]

In addition to the MR IVW method, we included MR Egger, weighted median, simple mode, and weighted mode estimators to further evaluate the relationships between brain structure measures and alcohol consumption behaviors. These complementary estimators help to assess the sensitivity of our results to different patterns of violations of instrumental variable assumptions. Consistency of results across MR methods strengthens an inference of causality[18] and employing these complementary methods facilitates an unbiased MR estimate.[18,53,54] The practice of using combinations of MR methods and examining the MR estimates across them has become critical because MR is now being used to evaluate the impact of complex exposures–such as global cortical thickness and neuropsychiatric endpoints–rather than better annotated biological phenotypes (e.g. circulating lipids, interleukin 6 levels, etc.).[18,19] In analyses evaluating complex traits, the exact biological mechanisms through which the variants impact the exposure are often unknown,[18,19] necessitating the use of these complementary MR methods.[18,19]

MR Egger extends the MR IVW method by not setting the linear regression intercept to zero, which allows the net horizontal pleiotropic MR estimate across all SNPs to be unbalanced or directional (i.e., some SNPs may be acting on the outcome via one or more pathways other than through the exposure).[52,55] The Instrument Strength Independent of Direct Effect (InSIDE) assumption on which MR Egger relies is a relaxed assumption compared to the strict MR assumption of no pleiotropy.[52,55] Although MR Egger returns unbiased estimates even if the assumption of no horizontal pleiotropy is violated for all SNPs; its effect estimates may be substantially less precise than MR IVW. Further, while the precision of the IVW estimate depends on the proportion of variance in the exposure explained by the genetic instruments (the R^2), MR Egger precision depends on the variability of the SNP-exposure associations.[52,55]

The weighted median estimator uses the median association of all available instrument SNPs, so that in order to return an unbiased effect estimate, only half of the SNPs need to be valid instruments (i.e., variants with no horizontal pleiotropy, no associations with confounders, and robust associations with the exposure). Furthermore, stronger SNPs contribute more towards the weighted median MR estimate, with the contribution of each SNP weighted by the inverse variance of its association with the outcome.[56]

The simple mode-based MR method clusters SNPs into groups based on similarity of MR associations and returns the MR estimate based on the cluster with the most SNPs. With this method, the MR estimate is unbiased as long as the SNPs within the largest cluster are valid instruments.[18] Weighted mode MR weights the contribution of each

© 2022 Mavromatis LA et al. *JAMA Psychiatry.*

variant to the clustering by the inverse variance of its outcome association. Assuming the most common MR instrument is consistent, the overall estimated MR relationship will be unbiased, even if all other instruments are invalid.[57]

MVMR: For our MVMR analysis, we used the multivariable extensions of IVW, MR Egger, and weighted median, and post-Lasso IVW methods to investigate the direct effect estimates of total cortical thickness on each of our drinking phenotypes and test the robustness of our MR estimates.[58-60] We also used multivariable extensions of the MR Egger intercept test[60] and the Cochran Q heterogeneity test[61] to evaluate heterogeneity in instrument effects.

Heterogeneity and pleiotropy corrections:

In order to evaluate the presence of potential heterogeneity and pleiotropy, which would violate the MR exclusion restriction assumption, we generated effect estimates after removing heterogenous instrument SNPs using MR PRESSO[62] when the Cochran Q heterogeneity test[63] yielded a P-value<.05. MR PRESSO implements an outlier test to identify heterogenous instruments, which we then removed and recalculated effect estimates using inverse variance weighted, MR Egger, weighted median, weighted mode, and simple mode estimators.

We also reported an MR Lasso[64] estimate for each exposure-outcome pair evaluated in this study. MR Lasso applies a lasso-type penalization to the direct effects of the instruments on the outcome disease. The so-called post-lasso estimate is obtained by performing IVW using only those instruments identified as valid with the tuning parameter specified at the default heterogeneity stopping rule.[64] As a further sensitivity analysis, we did not remove palindromic variants from the harmonized data for the MR Lasso estimates, which in cases where the Cochran Q heterogeneity test did not identify heterogeneous variants, led to some analyses where there were more variants included in the MR Lasso estimate than in the MR IVW estimate; however, they aligned in magnitude, direction, and statistical significance.

The presence of high influence SNPs among the variants in the GCT instrument would suggest potential horizontal pleiotropy for that variant that may violate the MR exclusion restriction assumption.[65] Therefore, to test the finding that GCT impacts alcoholic drinks consumed per week and binge drinking frequency, we performed a leave-one-out MR analysis to detect high influence SNPs.[65] We did not perform leave-one-out MR with rs2316766 removed because our post-Lasso IVW estimates showed that our findings were robust to the removal of this heterogenous SNP.

Steiger directionality test:

The Steiger directionality test is an important sensitivity analysis in bidirectional MR given the potential for reverse causality.[47] The Steiger directionality test is used to ascertain the directionality of a putative MR association, even when the underlying biology of the instrument variants is not understood.[47] We used the Steiger directionality test to infer the direction of causality for each of the brain structure-alcohol use analyses. While Steiger filtering performed during instrument harmonization compares the variance explained by individuals SNPs for the exposure and outcome datasets and removes SNPs if they explain more of the variance in the outcome than in the exposure,[47] the Steiger directionality test is statistical test for whether the assumption that the exposure causes the outcome is valid.[66] The Steiger directionality test compares the estimated variance explained in the exposure to the estimated variance explained in the outcome for all SNPs included in the MR analysis and tests whether the variance in the outcome is less than the variance in the exposure.[47] We set a Steiger directionality test P-value threshold of .05 to assess whether the inferred directionality of the MR analysis is true (i.e., P<.05 suggests a true causal direction) (**eTables**).

MR multiple testing correction procedures:

For our MR analyses, we accounted for multiple testing by defining significance at a false discovery rate (FDR) of .05. We performed separate FDR corrections for different subsets of MR analyses; FDR was calculated for 1) global cortical variables on drinking phenotypes, 2) drinking phenotypes on global cortical variables, 3) subcortical variables on drinking phenotypes, 4) drinking phenotypes on subcortical variables; 5) regional thickness variables on

binge drinking frequency, 6) AUD, and 7) alcoholic drinks consumed per week. These FDR calculations were made separately for each MR method (IVW, weighted median, post-lasso IVW, etc.).

Assessing the biological function of the global cortical thickness MR instrument:

Our primary bidirectional MR analysis revealed that a genetic liability for GCT negatively associated with alcohol use behaviors. While we used several complementary MR methods, in addition to MVMR models, to assess the robustness of these GCT findings—which is important in MR analyses where the biological function of the instrument SNPs are unknown[18,19]—we sought to evaluate the biological roles of the 6 SNPs comprising the GCT instrument via a gene-set enrichment analysis using gene-sets from the Gene Ontology biological process domain.[67] We used biomaRt[68] to identify genes located $\pm$100 kilobases of the 6 SNPs used for the GCT instrument in both single-variable and MVMR analyses. We then used the ENRICHR gene-set enrichment method (using default settings).[69]

Transcriptomic imputation (TWAS):

To perform transcriptomic imputation, we began by converting our GWAS summary statistics to FUSION-compatible formats with the mtag_munge.py script from LD Score.[70] Next, we used FUSION to perform transcriptomic imputation.[71] FUSION first identified cis-heritable genes located on autosomal chromosomes. For each of these genes, FUSION developed expression quantitative trait loci (eQTL)-based linear models to predict mRNA expression levels or splicing based on RNA-seq reference panels. We used the CommonMind Consortium's (CMC)[72] dorsolateral prefrontal cortex (DLPFC) RNA-seq expression (N = 452) and splicing (N = 452) reference panels, and Genotype-Tissue Expression version 7's (GTEx v7)[73] cortical RNA-seq expression reference panel (N = 136). FUSION tested multiple predictive models, including penalized several linear regression and Bayesian spare linear mixed models, and calculated the FUSION transcriptomic imputation test-statistic using the model that produced the highest out-of-sample R^2 value. We defined significant results at FDR=.05 based on the number of genes tested across all reference panels for a given alcohol use phenotype.

CELLECT cell-type enrichment analysis: methodological details and cell type nomenclature:

First, CELLEX calculated the expression specificity likelihood (ESμ) for each gene in each cell type after pre-processing and normalization. We then ran CELLECT with default settings using our alcohol-related GWAS summary statistics and the cortical data output by CELLEX. S-LD SCore (S-LDSC)[74] was used to calculate the degree to which SNP heritability for alcohol use behaviors was enriched in genes specifically expressed in a given cortical cell type. We defined significance at FDR=.05, calculated separately for each alcohol use behavior.

We used the Allen Brain Institute's Common Cell Type Nomenclature (CCN)[75] of 120 cell types to describe the cortical cell types included in our cell-type enrichment analyses. CCN incorporates broad cellular properties, anatomical information, and marker genes to produce cell-type names. We broadly focused our analyses on broad cellular categories with clear functional significance: excitatory cells (glutamatergic cells), inhibitory cells (GABAergic cells), and non-neuronal cells (astrocytes, oligodendrocytes, endothelial cells, microglia, and other glial cells). However, the cell type names we used also incorporated anatomical information about the cortical layer in which soma reside (1-6), and marker gene information defined by the maximal median counts per million among *ADARB2*, *PAX6, LAMP5, VIP, SST, PVALB, LINC00507, RORB, THEMIS, FEZF2, TYROBP, FGFR3, PDGFRA*, and *OPALIN*. A full explanation of the nomenclature used in this study is located on the Allen Brain Institute's website (https://portal.brain-map.org/explore/classes/nomenclature).

eResults

MR of regional cortical thickness on alcohol use behaviors:

© 2022 Mavromatis LA et al. *JAMA Psychiatry.*

Because we identified no relationships between regional cortical thickness phenotypes and alcohol use at FDR=.05, we conducted an exploratory analysis focused on nominal relationships (P<.05, **eTables 31-33**). A genetic liability for greater middle temporal cortical thickness (β=-3.74, CI [-6.69, -.80]) and entorhinal cortical thickness (β=-1.21, CI [-2.26, -.17]) were negatively associated with AUD. Genetic variants associated with the thickness of the isthmus cingulate (β=-.50, CI [-.84, -.16]), precuneus (β=-.90, CI [-1.67, -.13]), fusiform (β=.79, CI [.046, 1.53]), and insula (β=.49, CI [.0027, .98]) nominally associated with binge drinking frequency. Finally, our analyses identified nominal positive associations between genetic liabilities for inferior parietal (β=.36, CI [.028, .69]) and lateral orbitofrontal thickness (β=.39, CI [.008, .76]) and DPW and nominal negative associations between genetic liabilities for rostral middle frontal thickness (β=-.84, CI [-1.66, -.016]) and precuneus thickness (β=-.41, CI [-.81, -.005]) and DPW. The only regional cortical phenotype with a nominally significant unidirectional association with multiple alcohol use behaviors was precuneus thickness; however, these findings were not significant at FDR=.05. Ultimately, global cortical thickness's negative modulation of drinking behaviors remained our most robust MR finding.

MR of global cortical thickness on alcohol use in genetic males and females:

Our evaluation of genetically-predicted global cortical thickness and alcohol use in male and female samples yielded three significant results (P<.05): Greater genetic liability for cortical thickness was significantly associated with lower binge drinking frequency in males (β=-3.67, CI [-5.97, -1.37], P=.002) and females (β=-1.61, CI [-2.92, -.31], P=.015) and lower averaged alcohol consumed on a typical drinking day in females (β =-1.28, CI [-2.23, -.34], P=.008). There was no significant difference between the IVW effect estimate in male and female samples for binge drinking frequency (P=.13) or average alcohol consumed on a typical drinking day (P=.89). While our analysis was limited the unavailability of sex-specific cortical thickness datasets and limited sex-specific alcohol use datasets, we find no evidence that the effect of global cortical thickness on alcohol use is primarily driven by males or females.

Leave-one-out analysis shows no evidence of high influence SNPs among variants comprising the GCT instrument:

Presented in **eFigure 4** and **eTable 36**, leave-one-out MR analysis results showed significant (P<.05) and consistent MR IVW estimates across the 5 models that, in turn, remove one of the GCT instrument variants. These results support the validity of the GCT instrument.[65]

Gene set enrichment analysis reveals that genes near GCT instrument variants are implicated in biological processes related to brain function:

The top Gene Ontology (GO) gene set enriched with the GCT instrument genes was related to axonal transport of mitochondrion (GO ID: 0019896, P=9.85×10^{-5}). Other top gene-sets included broad axonal transport (GO ID: 009830, P=.001); positive regulation of protein localization to the synapse (GO ID: 0031113, P=.007); and cellular response to brain derived-neurotrophic factor stimulus (GO ID: 1990416, P=.007). Notably, it has been suggested that cortical thickness reflects not only size, density, and arrangement of neurons and nerve fibers, but also the remodeling of axons and dendrites.[76,77] Given the fundamental importance of axonal transport to synaptic functioning,[78] our gene set enrichment analyses suggest the GCT instrument SNPs may impact cortical thickness via their role in axonal transport.

eDiscussion

Comparison of results from Logtenberg et al., (2021) bidirectional MR of overall subcortical volumes and alcohol use and our bidirectional MR of left/right subcortical volumes and alcohol use:

To our knowledge, Logtenberg et al., (2021)[79] is the only other study that has investigated interactions between brain structure and alcohol use behaviors using bidirectional MR. In that study, the authors focused on bidirectional associations between DPW and AUD and subcortical volumes. Logtenberg et al. identified negative associations

© 2022 Mavromatis LA et al. *JAMA Psychiatry.*

between genetically-predicted AUD and whole amygdala volume and between genetically-predicted AUD and whole hippocampal volume. The authors identified no relationships between subcortical exposures and alcohol-related outcomes.

Discrepancies in data sources and methodology likely explain the differences in the subcortical findings between Logtenberg et al., (2021) and the present study. While the present study defined significance at a conservative FDR of .05, Logtenberg et al. did not implement multiple testing correction. Therefore, while both studies identify a negative relationship between genetically-predicted alcohol use and amygdala volume at $P<.05$, the negative relationship between right amygdala volume and alcohol use that we identify does not surpass our multiple-testing threshold. Had Logtenberg et al., (2021) implemented a similar multiple testing correction, the relationship they identified may not have been considered significant. Similarly, while this study identifies no effect of alcohol use on hippocampal volume, the relationship Logtenberg et al. identified would have been considered a null effect had an FDR-correction been implemented.

With respect to relationships between subcortical exposures and alcohol-related outcomes, our study identifies some evidence for a positive relationship between right pallidum volume and alcohol use behaviors. Logtenberg et al. identified no evidence of such an effect. Our hemisphere-specific result could have been obscured by Logetenberg et al.'s consideration of average subcortical volumes across hemispheres. Additionally, the alcohol use behavior most robustly associated with right pallidum volume in our study was binge drinking frequency, a drinking behavior that Logtenberg et al. did not analyze.

Other sources of discrepancies between our study and Logtenberg et al., could have been different procedures for instrument selection and/or subcortical data sample size. For instance, Logtenberg et al., used a sample (N=50,290) that was more than twice as large as ours (N=19,629), while we opted for a GWAS that considered left/right functional asymmetry. Future studies with larger left/right subcortical volume GWAS data should attempt to verify the evidence of hemispheric asymmetry suggested by the present study.

eReferences

1. Bycroft C, Freeman C, Petkova D, et al. The UK Biobank resource with deep phenotyping and genomic data. *Nature.* 2018;562(7726):203-209.
2. Walters RK, Polimanti R, Johnson EC, et al. Transancestral GWAS of alcohol dependence reveals common genetic underpinnings with psychiatric disorders. *Nature neuroscience.* 2018;21(12):1656-1669.
3. Association AP. *Diagnostic and Statistical Manual of Mental Disorders.* 4 ed. Washington, DC: American Psychiatric Association; 1994.
4. Association AP. *Diagnostic and Statistical Manual of Mental Disorders.* 5 ed. Arlington, VA: American Psychiatric Association; 2013.
5. Mackey S, Allgaier N, Chaarani B, et al. Mega-analysis of gray matter volume in substance dependence: general and substance-specific regional effects. *American Journal of Psychiatry.* 2019;176(2):119-128.
6. Satizabal CL, Adams HHH, Hibar DP, et al. Genetic architecture of subcortical brain structures in 38,851 individuals. *Nature Genetics.* 2019;51(11):1624-1636.
7. Welch KA. Alcohol consumption and brain health. *BMJ (Clinical research ed).* 2017;357:j2645-j2645.
8. Brouwer RM, Klein M, Grasby KL, et al. Genetic variants associated with longitudinal changes in brain structure across the lifespan. *Nature Neuroscience.* 2022;25(4):421-432.
9. Davey Smith G, Hemani G. Mendelian randomization: genetic anchors for causal inference in epidemiological studies. *Human molecular genetics.* 2014;23(R1):R89-R98.
10. Smith GD. Use of genetic markers and gene-diet interactions for interrogating population-level causal influences of diet on health. *Genes & nutrition.* 2011;6(1):27-43.
11. Smith GD, Ebrahim S. 'Mendelian randomization': can genetic epidemiology contribute to understanding environmental determinants of disease? *Int J Epidemiol.* 2003;32(1):1-22.

© 2022 Mavromatis LA et al. *JAMA Psychiatry.*

12. Davies NM, Holmes MV, Smith GD. Reading Mendelian randomisation studies: a guide, glossary, and checklist for clinicians. *Bmj.* 2018;362.
13. Eng MY, Luczak SE, Wall TL. ALDH2, ADH1B, and ADH1C genotypes in Asians: a literature review. *Alcohol Res Health.* 2007;30(1):22-27.
14. Zhu C, Chen Q, Si W, Li Y, Chen G, Zhao Q. Alcohol Use and Depression: A Mendelian Randomization Study From China. *Frontiers in Genetics.* 2020;11.
15. Lawlor DA, Benn M, Zuccolo L, et al. ADH1B and ADH1C genotype, alcohol consumption and biomarkers of liver function: findings from a Mendelian randomization study in 58,313 European origin Danes. *PLoS One.* 2014;9(12):e114294.
16. Holmes MV, Dale CE, Zuccolo L, et al. Association between alcohol and cardiovascular disease: Mendelian randomisation analysis based on individual participant data. *Bmj.* 2014;349:g4164.
17. Polimanti R, Gelernter J. ADH1B: From alcoholism, natural selection, and cancer to the human phenome. *Am J Med Genet B Neuropsychiatr Genet.* 2018;177(2):113-125.
18. Hemani G, Zheng J, Elsworth B, et al. The MR-Base platform supports systematic causal inference across the human phenome. *Elife.* 2018;7.
19. Wootton RE, Jones HJ, Sallis HM. Mendelian randomisation for psychiatry: how does it work, and what can it tell us? *Molecular Psychiatry.* 2022;27(1):53-57.
20. Touchette JC, Lee AM. Assessing alcohol and nicotine co-consumption in mice. *Oncotarget.* 2017;8(4):5684-5685.
21. Grant BF, Hasin DS, Chou SP, Stinson FS, Dawson DA. Nicotine Dependence and Psychiatric Disorders in the United States: Results From the National Epidemiologic Survey on Alcohol and RelatedConditions. *Archives of General Psychiatry.* 2004;61(11):1107-1115.
22. Marees AT, Smit DJA, Ong JS, et al. Potential influence of socioeconomic status on genetic correlations between alcohol consumption measures and mental health. *Psychol Med.* 2020;50(3):484-498.
23. Liu M, Jiang Y, Wedow R, et al. Association studies of up to 1.2 million individuals yield new insights into the genetic etiology of tobacco and alcohol use. *Nature genetics.* 2019;51(2):237-244.
24. Elsworth B, Mitchell, R, Raistrick, CA, Paternoster, L, Hemani, G, Gaunt, TR MRC IEU UK Biobank GWAS pipeline version 1. 2017. doi: https://doi.org/10.5523/bris.2fahpksont1zi26xosyamqo8rr. Accessed Feb 2, 2019.
25. Sanderson E, Davey Smith G, Windmeijer F, Bowden J. An examination of multivariable Mendelian randomization in the single-sample and two-sample summary data settings. *Int J Epidemiol.* 2019;48(3):713-727.
26. Burgess S, Thompson SG. Multivariable Mendelian randomization: the use of pleiotropic genetic variants to estimate causal effects. *Am J Epidemiol.* 2015;181(4):251-260.
27. Rosoff DB, Yoo J, Lohoff FW. Smoking is significantly associated with increased risk of COVID-19 and other respiratory infections. *Communications Biology.* 2021;4(1):1230.
28. Richardson TG, Sanderson E, Palmer TM, et al. Evaluating the relationship between circulating lipoprotein lipids and apolipoproteins with risk of coronary heart disease: A multivariable Mendelian randomisation analysis. *PLOS Medicine.* 2020;17(3):e1003062.
29. Tylee DS, Lee YK, Wendt FR, et al. An Atlas of Genetic Correlations and Genetically Informed Associations Linking Psychiatric and Immune-Related Phenotypes. *JAMA Psychiatry.* 2022.
30. Davies NM, Hill WD, Anderson EL, Sanderson E, Deary IJ, Davey Smith G. Multivariable two-sample Mendelian randomization estimates of the effects of intelligence and education on health. *Elife.* 2019;8.
31. Carter AR, Sanderson E, Hammerton G, et al. Mendelian randomisation for mediation analysis: current methods and challenges for implementation. *Eur J Epidemiol.* 2021;36(5):465-478.
32. Sanderson E. Multivariable Mendelian Randomization and Mediation. *Cold Spring Harb Perspect Med.* 2021;11(2).

© 2022 Mavromatis LA et al. *JAMA Psychiatry.*

33. Schizophrenia Working Group of the Psychiatric Genomics C. Biological insights from 108 schizophrenia-associated genetic loci. *Nature.* 2014;511(7510):421-427.
34. Stahl EA, Breen G, Forstner AJ, et al. Genome-wide association study identifies 30 loci associated with bipolar disorder. *Nat Genet.* 2019;51(5):793-803.
35. Howard DM, Adams MJ, Clarke T-K, et al. Genome-wide meta-analysis of depression identifies 102 independent variants and highlights the importance of the prefrontal brain regions. *Nature neuroscience.* 2019;22(3):343-352.
36. Demontis D, Walters RK, Martin J, et al. Discovery of the first genome-wide significant risk loci for attention deficit/hyperactivity disorder. *Nature Genetics.* 2019;51(1):63-75.
37. Jansen IE, Savage JE, Watanabe K, et al. Genome-wide meta-analysis identifies new loci and functional pathways influencing Alzheimer's disease risk. *Nature Genetics.* 2019;51(3):404-413.
38. Lee JJ, Wedow R, Okbay A, et al. Gene discovery and polygenic prediction from a genome-wide association study of educational attainment in 1.1 million individuals. *Nature genetics.* 2018;50(8):1112-1121.
39. Ivy AS, Rex CS, Chen Y, et al. Hippocampal dysfunction and cognitive impairments provoked by chronic early-life stress involve excessive activation of CRH receptors. *J Neurosci.* 2010;30(39):13005-13015.
40. Wang X-D, Su Y-A, Wagner KV, et al. Nectin-3 links CRHR1 signaling to stress-induced memory deficits and spine loss. *Nature Neuroscience.* 2013;16(6):706-713.
41. Nievergelt CM, Maihofer AX, Klengel T, et al. International meta-analysis of PTSD genome-wide association studies identifies sex- and ancestry-specific genetic risk loci. *Nat Commun.* 2019;10(1):4558.
42. Coleman JRI, Peyrot WJ, Purves KL, et al. Genome-wide gene-environment analyses of major depressive disorder and reported lifetime traumatic experiences in UK Biobank. *Mol Psychiatry.* 2020;25(7):1430-1446.
43. Pasman JA, Verweij KJH, Gerring Z, et al. GWAS of lifetime cannabis use reveals new risk loci, genetic overlap with psychiatric traits, and a causal effect of schizophrenia liability. *Nature Neuroscience.* 2018;21(9):1161-1170.
44. Burgess S, Davies NM, Thompson SG. Bias due to participant overlap in two-sample Mendelian randomization. *Genet Epidemiol.* 2016;40(7):597-608.
45. Minelli C, Del Greco MF, van der Plaat DA, Bowden J, Sheehan NA, Thompson J. The use of two-sample methods for Mendelian randomization analyses on single large datasets. *Int J Epidemiol.* 2021;50(5):1651-1659.
46. Yavorska OO, Burgess S. MendelianRandomization: an R package for performing Mendelian randomization analyses using summarized data. *International journal of epidemiology.* 2017;46(6):1734-1739.
47. Hemani G, Tilling K, Davey Smith G. Orienting the causal relationship between imprecisely measured traits using GWAS summary data. *PLoS genetics.* 2017;13(11):e1007081.
48. Burgess S, Thompson SG, Collaboration CCG. Avoiding bias from weak instruments in Mendelian randomization studies. *International journal of epidemiology.* 2011;40(3):755-764.
49. Sanderson E, Spiller W, Bowden J. Testing and Correcting for Weak and Pleiotropic Instruments in Two-Sample Multivariable Mendelian Randomisation. *bioRxiv (preprint).* 2020:2020.2004.2002.021980.
50. Liu J, Au Yeung SL, Kwok MK, et al. The effect of liver enzymes on adiposity: a Mendelian randomization study. *Scientific Reports.* 2019;9(1):16792.
51. Burgess S, Butterworth A, Thompson SG. Mendelian randomization analysis with multiple genetic variants using summarized data. *Genet Epidemiol.* 2013;37(7):658-665.
52. Bowden J, Smith GD, Burgess S. Mendelian randomization with invalid instruments: effect estimation and bias detection through Egger regression. *International Journal of Epidemiology.* 2015;44(2):512-525.

53. Sanderson E, Glymour MM, Holmes MV, et al. Mendelian randomization. *Nature Reviews Methods Primers.* 2022;2(1):6.
54. Davey Smith G, Hemani G. Mendelian randomization: genetic anchors for causal inference in epidemiological studies. *Hum Mol Genet.* 2014;23(R1):R89-98.
55. Davey Smith G, Bowden J, Del Greco M F, Minelli C, Thompson JR, Sheehan NA. Assessing the suitability of summary data for two-sample Mendelian randomization analyses using MR-Egger regression: the role of the I2 statistic. *International Journal of Epidemiology.* 2016;45(6):1961-1974.
56. Bowden J, Davey Smith G, Haycock PC, Burgess S. Consistent Estimation in Mendelian Randomization with Some Invalid Instruments Using a Weighted Median Estimator. *Genet Epidemiol.* 2016;40(4):304-314.
57. Hartwig FP, Davey Smith G, Bowden J. Robust inference in summary data Mendelian randomization via the zero modal pleiotropy assumption. *Int J Epidemiol.* 2017;46(6):1985-1998.
58. Burgess S, Thompson SG. Multivariable Mendelian Randomization: The Use of Pleiotropic Genetic Variants to Estimate Causal Effects. *American Journal of Epidemiology.* 2015;181(4):251-260.
59. Grant AJ, Burgess S. Pleiotropy robust methods for multivariable Mendelian randomization. *Stat Med.* 2021;40(26):5813-5830.
60. Rees JMB, Wood AM, Burgess S. Extending the MR-Egger method for multivariable Mendelian randomization to correct for both measured and unmeasured pleiotropy. *Stat Med.* 2017;36(29):4705-4718.
61. Sanderson E, Davey Smith G, Windmeijer F, Bowden J. An examination of multivariable Mendelian randomization in the single-sample and two-sample summary data settings. *International Journal of Epidemiology.* 2018;48(3):713-727.
62. Verbanck M, Chen C-y, Neale B, Do R. Detection of widespread horizontal pleiotropy in causal relationships inferred from Mendelian randomization between complex traits and diseases. *Nature genetics.* 2018;50(5):693-698.
63. Bowden J, Del Greco M F, Minelli C, et al. Improving the accuracy of two-sample summary-data Mendelian randomization: moving beyond the NOME assumption. *International journal of epidemiology.* 2019;48(3):728-742.
64. Rees JMB, Wood AM, Dudbridge F, Burgess S. Robust methods in Mendelian randomization via penalization of heterogeneous causal estimates. *PLoS One.* 2019;14(9):e0222362.
65. Hemani G, Bowden J, Davey Smith G. Evaluating the potential role of pleiotropy in Mendelian randomization studies. *Human Molecular Genetics.* 2018;27(R2):R195-R208.
66. Hemani G, Zheng J, Elsworth B, et al. The MR-Base platform supports systematic causal inference across the human phenome. *elife.* 2018;7:e34408.
67. Ashburner M, Ball CA, Blake JA, et al. Gene Ontology: tool for the unification of biology. *Nature Genetics.* 2000;25(1):25-29.
68. Durinck S, Spellman PT, Birney E, Huber W. Mapping identifiers for the integration of genomic datasets with the R/Bioconductor package biomaRt. *Nat Protoc.* 2009;4(8):1184-1191.
69. Chen EY, Tan CM, Kou Y, et al. Enrichr: interactive and collaborative HTML5 gene list enrichment analysis tool. *BMC Bioinformatics.* 2013;14(1):128.
70. Bulik-Sullivan BK, Loh P-R, Finucane HK, et al. LD Score regression distinguishes confounding from polygenicity in genome-wide association studies. *Nature genetics.* 2015;47(3):291-295.
71. Gusev A, Ko A, Shi H, et al. Integrative approaches for large-scale transcriptome-wide association studies. *Nat Genet.* 2016;48(3):245-252.
72. Fromer M, Roussos P, Sieberts SK, et al. Gene expression elucidates functional impact of polygenic risk for schizophrenia. *Nature neuroscience.* 2016;19(11):1442-1453.
73. Consortium G. Human genomics. The Genotype-Tissue Expression (GTEx) pilot analysis: multitissue gene regulation in humans. *Science.* 2015;348(6235):648-660.

© 2022 Mavromatis LA et al. *JAMA Psychiatry.*

74. Finucane HK, Bulik-Sullivan B, Gusev A, et al. Partitioning heritability by functional annotation using genome-wide association summary statistics. *Nature genetics.* 2015;47(11):1228-1235.
75. Miller JA, Gouwens NW, Tasic B, et al. Common cell type nomenclature for the mammalian brain. *Elife.* 2020;9:e59928.
76. Beckwith T, Cecil K, Altaye M, et al. Reduced gray matter volume and cortical thickness associated with traffic-related air pollution in a longitudinally studied pediatric cohort. *PLOS ONE.* 2020;15(1):e0228092.
77. Paus T, Keshavan M, Giedd JN. Why do many psychiatric disorders emerge during adolescence? *Nat Rev Neurosci.* 2008;9(12):947-957.
78. Guedes-Dias P, Holzbaur ELF. Axonal transport: Driving synaptic function. *Science (New York, NY).* 2019;366(6462):eaaw9997.
79. Logtenberg E, Overbeek MF, Pasman JA, et al. Investigating the causal nature of the relationship of subcortical brain volume with smoking and alcohol use. *The British Journal of Psychiatry.* 2021:1-9.

eFigures

eFigure 1. Mendelian randomization model and assumptions

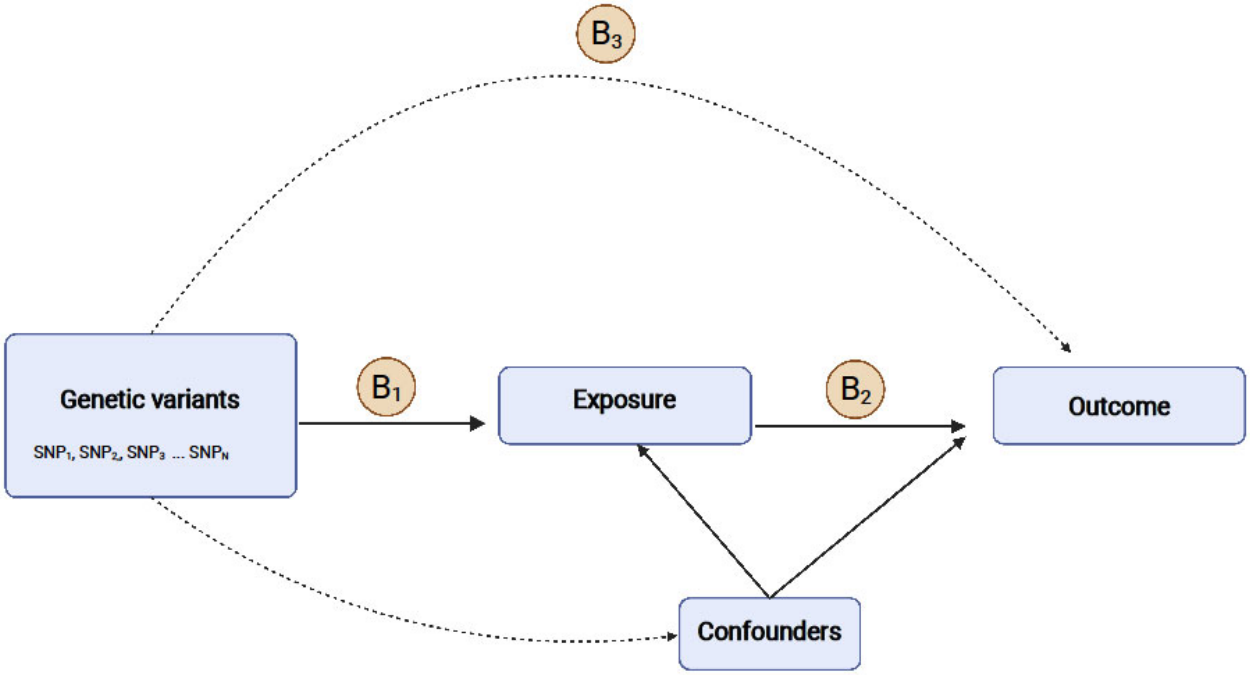

B_2 is the genetic association of interest, estimated by B_2=B_1/ B_3. B_1 and B_3 are the associations of the genetic variants with the exposure and the outcome. MR assumes that the genetic variants comprising the instrument for the exposure only impact the outcome of interest via the exposure and not directly, or via confounders (dotted lines).[53]

eFigure 2. Multivariable Mendelian randomization: methodological overview

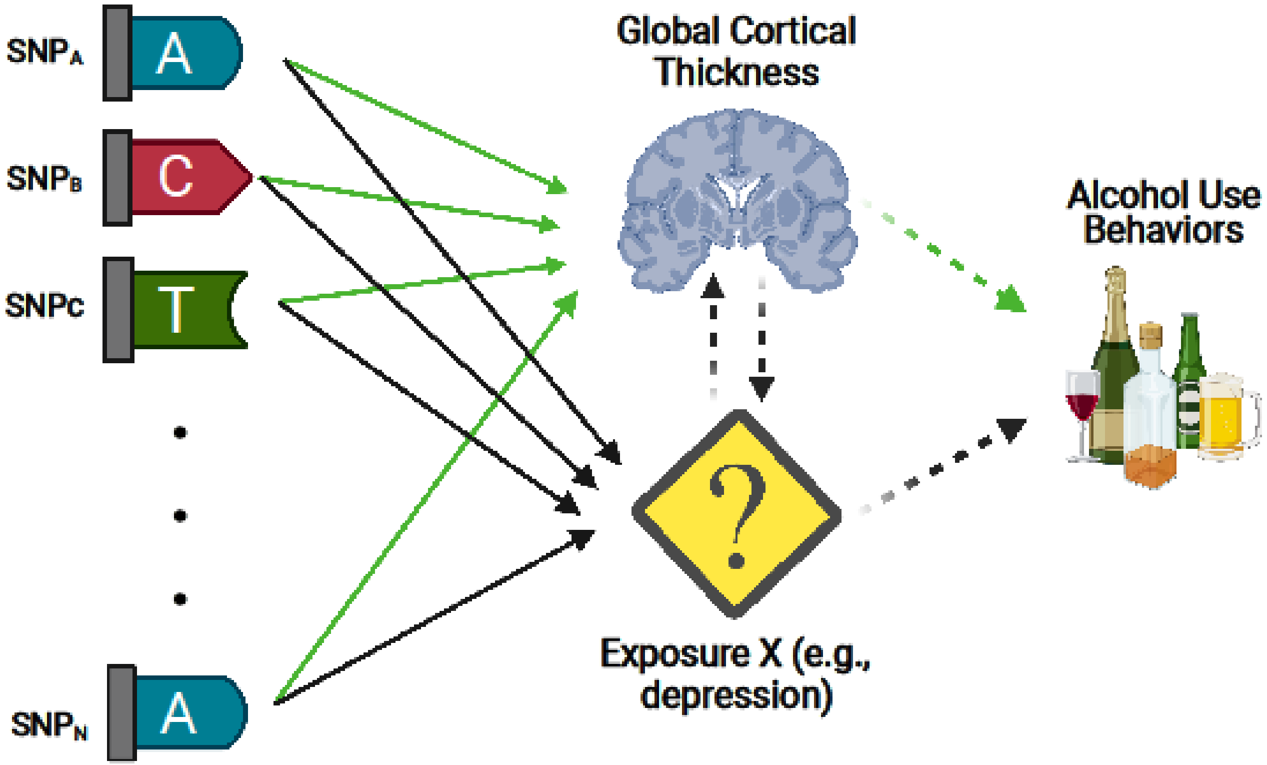

Multivariable Mendelian randomization (MVMR) extends traditional MR to quantify the total, indirect, and direct effects of exposures on outcomes. Our MVMR models use genetic instruments (SNPs) associated with both global cortical thickness and a potential mediator/confounder (exposure X) to estimate the direct effect of global cortical thickness on alcohol use behaviors. Green arrows represent the direct effect.

© 2022 Mavromatis LA et al. *JAMA Psychiatry.*

eFigure 3. Possible pleiotropic models explaining relationships between SNP instruments, global cortical thickness, and alcohol use behaviors

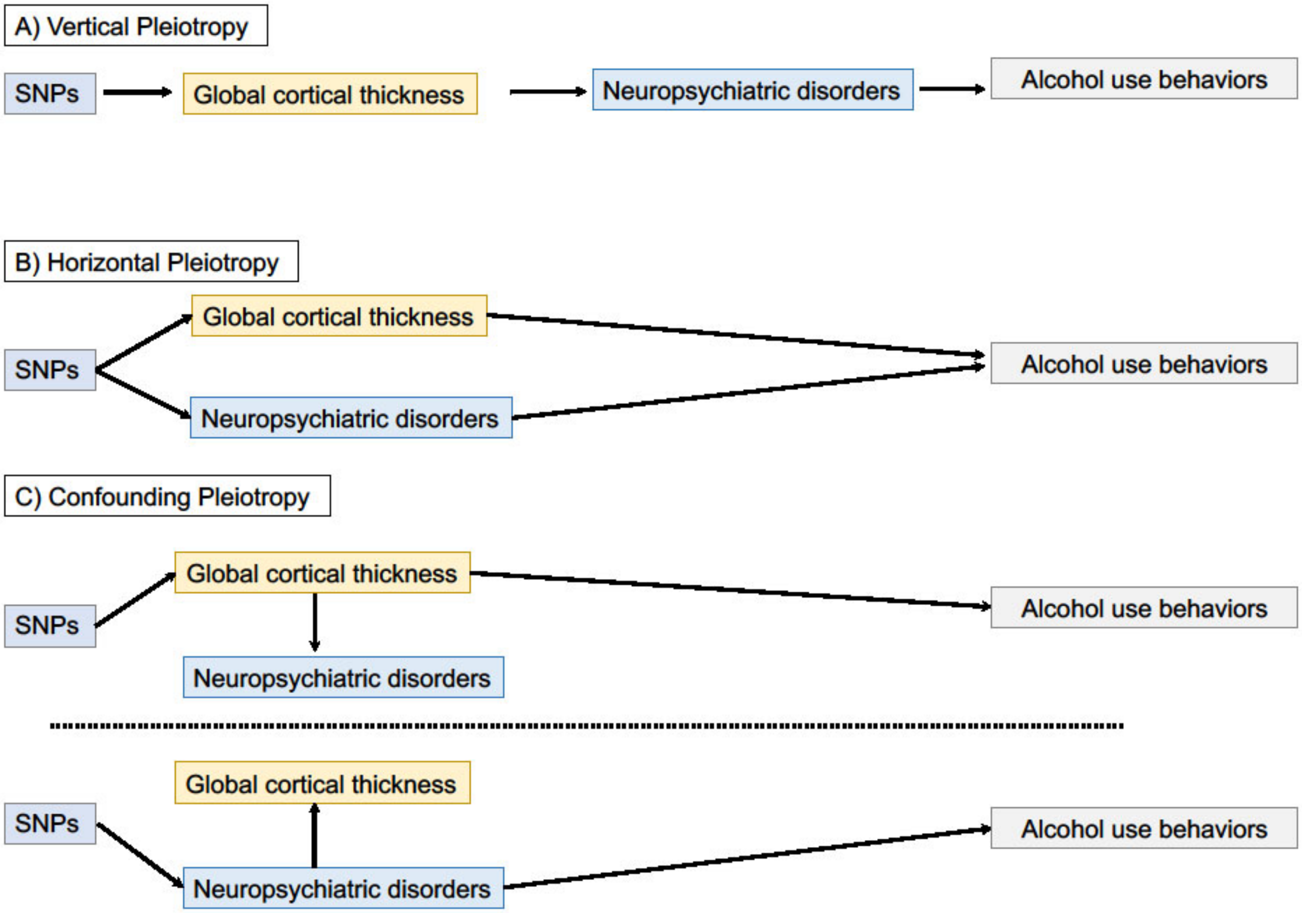

(A) depicts potential vertical pleiotropy in a single-variable MR study scenario. Here SNPs (single nucleotide polymorphisms) act as instruments for global cortical thickness (GCT); they would demonstrate vertical pleiotropy if the genetic effects on their respective outcomes are mediated by their corresponding downstream impact (i.e., GCT SNP instruments impacting a neuropsychiatric disorder, which, in turn, impacts alcohol use). (B) depicts potential horizontal pleiotropy where SNPs have an impact on alcohol use behaviors via their associations with GCT *and* neuropsychiatric disorders lacking any downstream mediation. Confounding pleiotropy is depicted in (C): SNPs associated with GCT may impact alcohol use behaviors due to their impact on neuropsychiatric disorders; however, the genetic propensity for GCT has no direct impact on alcohol use behaviors (or vice versa).

© 2022 Mavromatis LA et al. *JAMA Psychiatry.*

eFigure 4. Results of leave-one-out analyses of GCT on drinks per week and binge drinking

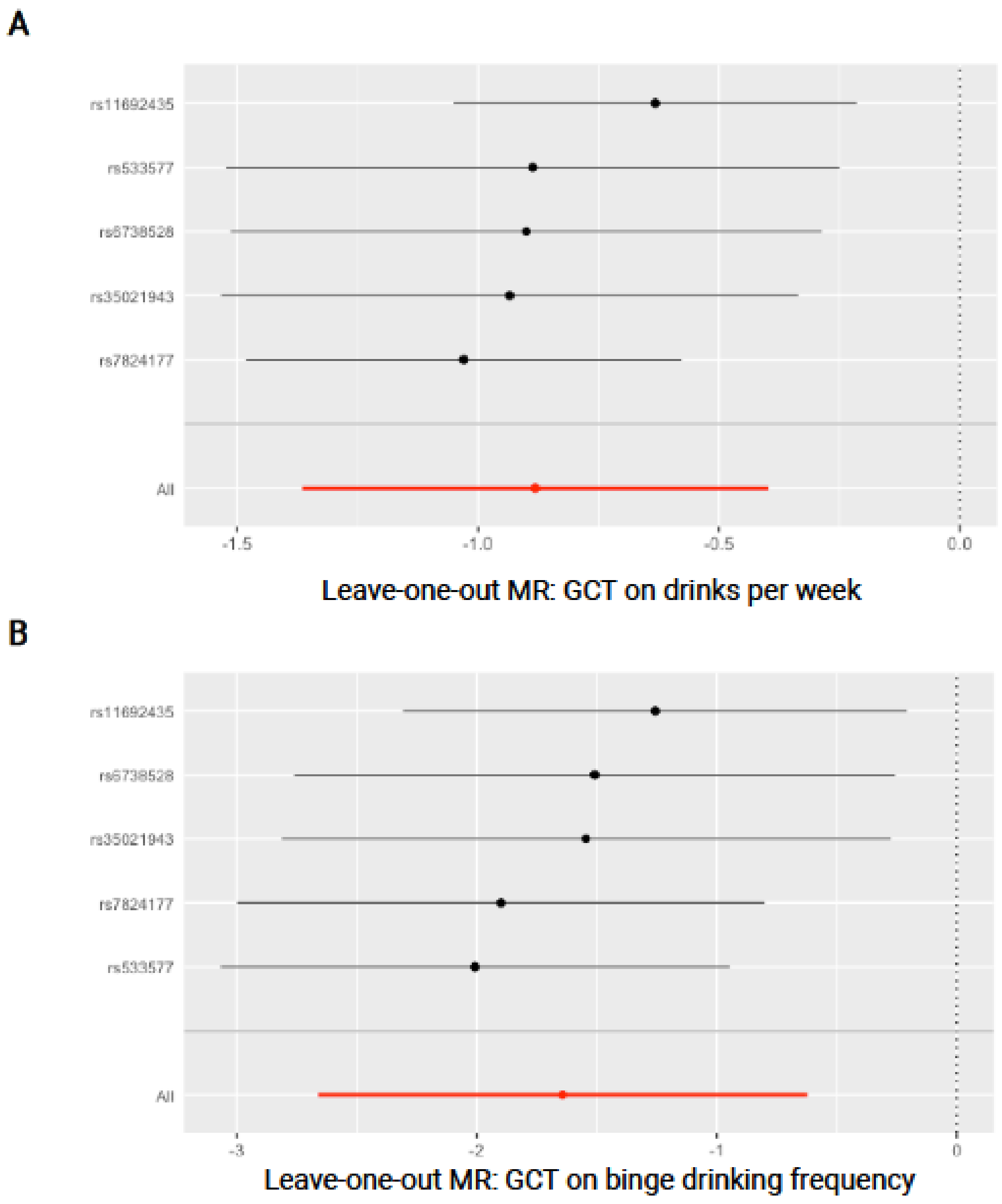

Panel (A) depicts the results of leave-one-out MR analyses of GCT on drinks per week and panel (B) depicts the results for the leave-one-out analyses of GCT on binge drinking. The x-axes are the magnitude of the inverse variance weighted (IVW) Mendelian randomization estimates (β) after removal of one of the GCT instrument variants. The y-axes indicate the specific GCT instrument variant removed for the resulting IVW estimate. “All” denotes the standard post-Lasso IVW estimate. All estimates are significant (P<.05). **eTable 36** contains full results.

© 2022 Mavromatis LA et al. *JAMA Psychiatry.*

eTable 1. STROBE-MR reporting guidelines

Relevant section	Item	Instruction	Location addressed
TITLE and ABSTRACT	TITLE and ABSTRACT	Indicate Mendelian randomization as the study's design in the title and/or the abstract.	Title and abstract
INTRODUCTION	Background	Explain the scientific background and rationale for the reported study. Is causality between exposure and outcome plausible? Justify why MR is a helpful method to address the study question.	Introduction
	Objectives	State specific objectives clearly, including pre-specified causal hypotheses (if any).	Introduction
METHODS	Study design and data sources	Present key elements of study design early in the paper. Consider including a table listing sources of data for all phases of the study. For each data source contributing to the analysis, describe the following:<br>a) Describe the study design and the underlying population from which it was drawn.<br>Describe also the setting, locations, and relevant dates, including periods of recruitment, exposure, follow-up, and data collection, if available.<br>b) Give the eligibility criteria, and the sources and methods of selection of participants.<br>c) Explain how the analyzed sample size was arrived at.<br>d) Describe measurement, quality and selection of genetic variants.<br>e) For each exposure, outcome and other relevant variables, describe methods of assessment and, in the case of diseases, the diagnostic criteria used.<br>f) Provide details of ethics committee approval and participant informed consent, if relevant.	a) Introduction, Methods, eMethods, Figure 1, eTable 2<br>b) Methods, eMethods<br>c) Methods, eMethods<br>d) Methods, eMethods<br>e) Methods, eMethods<br>f) Methods, eMethods
	Assumptions	Explicitly state assumptions for the main analysis (e.g. relevance, exclusion, independence, homogeneity) as well assumptions for any additional or sensitivity analysis.	Methods, eMethods
	Statistical methods: main analysis	Describe statistical methods and statistics used.<br>a) Describe how quantitative variables were handled in the analyses (i.e., scale, units, model).<br>b) Describe the process for identifying genetic variants and weights to be included in the analyses (i.e, independence and model). Consider a flow diagram.<br>c) Describe the MR estimator, e.g. two-stage least squares, Wald ratio, and related statistics.<br>Detail the included covariates and, in case of two-sample MR, whether the same covariate set was used for adjustment in the two samples.<br>d) Explain how missing data were addressed.<br>e) If applicable, say how multiple testing was dealt with.	a) Methods, eMethods<br>b) Methods, eMethods<br>c) Methods, eMethods<br>d) Methods, eMethods<br>e) Methods, eMethods
	Assessment of assumptions	Describe any methods used to assess the assumptions or justify their validity	Methods, eMethods
	Sensitivity analyses	Describe any sensitivity analyses or additional analyses performed.	Methods, eMethods
	Software and pre-registration	a) Name statistical software and package(s), including version and settings used; b) State whether the study protocol and details were pre-registered (as well as when and where).	Methods, eMethods

© 2022 Mavromatis LA et al. *JAMA Psychiatry.*

RESULTS	Descriptive data	a) Report the numbers of individuals at each stage of included studies and reasons for exclusion. Consider use of a flow-diagram.<br>b) Report summary statistics for phenotypic exposure(s), outcome(s) and other relevant variables (e.g. means, standard deviations, proportions).<br>c) If the data sources include meta-analyses of previous studies, provide the number of studies, their reported ancestry, if available, and assessments of heterogeneity across these studies. Consider using a supplementary table for each data source.<br>d) For two-sample Mendelian randomization:<br>i. Provide information on the similarity of the genetic variant-exposure associations between the exposure and outcome samples.<br>ii. Provide information on extent of sample overlap between the exposure and outcome data sources.	a) Methods, eMethods<br>b) Methods, eMethods, eTable 2<br>c) Methods, eMethods, eTable 2<br>d) Methods, eMethods<br>i. eMethods and eTables (instrument, harmonized, and results eTables)<br>ii. eMethods
	Main results	a) Report the associations between genetic variant and exposure, and between genetic variant and outcome, preferably on an interpretable scale (e.g. comparing 25th and 75th percentile of allele count or genetic risk score, if individual-level data available).<br>b) Report causal effect estimate between exposure and outcome, and the measures of uncertainty from the MR analysis. Use an intuitive scale, such as odds ratio, or relative risk, per standard deviation difference.<br>c) If relevant, consider translating estimates of relative risk into absolute risk for a meaningful time-period.<br>d) Consider any plots to visualize results (e.g. forest plot, scatterplot of associations between genetic variants and outcome versus between genetic variants and exposure).	a) N/A, no individual level data available<br>b) Results, eResults, eTables<br>c) N/A<br>d) Figure 2, eFigure 4
	Assessment of assumptions	a) Assess the validity of the assumptions.<br>b) Report any additional statistics (e.g., assessments of heterogeneity, such as I2, Q statistic)	a) Methods, Results, eMethods, eTables<br>b) eTables
	Sensitivity and additional analyses	a) Use sensitivity analyses to assess the robustness of the main results to violations of the assumptions.<br>b) Report results from other sensitivity analyses (e.g., replication study with different dataset, analyses of subgroups, validation of instrument(s), simulations, etc.).<br>c) Report any assessment of direction of causality (e.g., bidirectional MR).<br>d) When relevant, report and compare with estimates from non-MR analyses.<br>e) Consider any additional plots to visualize results (e.g., leave-one-out analyses).	a) Results, eTables<br>b) Results, eResults, eTables<br>c) Results, eResults, eTables<br>d) Introduction, Discussion<br>e) eFigure 4
DISCUSSION	Key results	Summarize key results with reference to study objectives.	Discussion
	Limitations	Discuss limitations of the study, taking into account the validity of the MR assumptions, other sources of potential bias, and imprecision. Discuss both direction and magnitude of any potential bias, and any efforts to address them.	Methods, Discussion, eMethods
	Interpretation	a) Give a cautious overall interpretation of results considering objectives and limitations.<br>Compare with results from other relevant studies.<br>b) Discuss underlying biological mechanisms that could be modelled by using the genetic variants to assess the relationship between the exposure and the outcome.<br>c) Discuss whether the results have clinical or policy relevance, and whether interventions could have the same size effect.	a) Results, Discussion<br>b) Results, Discussion<br>c) Abstract, Discussion
	Generalizability	Discuss the generalizability of the study results (a) to other populations (i.e. external validity),<br>(b) across other exposure periods/timings, and (c) across other levels of exposure.	a) Discussion<br>b) Discussion<br>c) N/A
OTHER INFORMATION	Funding	Give the source of funding and the role of the funders for the present study and, if applicable, for the original study or studies on which the present article is based.	Acknowledgements

© 2022 Mavromatis LA et al. *JAMA Psychiatry.*

	Data and data sharing	Present data used to perform all analyses or report where and how the data can be accessed. State whether statistical code is publicly accessible and if so, where.	Methods, eTables, References, eReferences, Additional Information
	Conflicts of Interest	All authors should declare all potential conflicts of interest.	Acknowledgements

eTable 2. Demographics of GWAS cohorts featured in single-variable MR analyses

Target trait	Source	Ancestry	Mean age (S.D.)	Percent female	N	Additional information
Cortical thickness and surface area (global and regional)	Enhancing Neuroimaging Genetics through Meta-Analysis Consortium (ENIGMA)--Genetics Working Group. Meta-analyis of 49 European ancestry ENIGMA cohorts (N=23,909) and a UK Biobank cohort (N=10,083)	~94% European ancestry	~40 +/- 8 years old	52%	33,709	https://www.science.org/doi/10.1126/science.aay6690
Hemispheric subcortical volumes	UK Biobank MRI data--2017 and 2018 releases	~100% European ancestry (British)	~63 +/- 7 years old	52%	19,629	https://www.nature.com/articles/s41588-019-0516-6#MOESM3
Subcortical volumes (combined hemispheres)	ENIGMA Wave 2	~94% European ancestry	~51 +/- 7	53%	30,175	https://enigma.ini.usc.edu/research/download-enigma-gwas-results/
Longitudinal changes in cortical and subcortical brain structures	ENIGMA Plasticity Working group	~100% European ancestry	~42 +/- 5 years old	49%	15,640	https://enigma.ini.usc.edu/research/download-enigma-gwas-results/
Alcoholic drinks consumed per week	Meta-analysis conducted by the GWAS & Sequencing Consortium of Alcohol and Nicotine use (GSCAN)	~100% European ancestry	Not reported, age included as covariate in GWAS	52%	537,349	https://www.ncbi.nlm.nih.gov/pmc/articles/PMC6358542/

© 2022 Mavromatis LA et al. *JAMA Psychiatry.*

Alcohol use disorder	Meta-analysis conducted by the Substance Use Disorders working group of the Psychiatric Genomics Consortium (PGC-SUD) -- unrelated individuals from 14 case/control studies, 9 family-based studies and 5 additional cohorts	~100% European ancestry	"Ages fully distributed across the lifespan for adults"	52%	29,502	https://www.ncbi.nlm.nih.gov/pmc/articles/PMC6430207/#SD3
Binge drinking frequency	A Neale Lab analysis of the UK Biobank	UK Biobank is ~95% European ancestry (Constantinescu et al., 2022)	58 +/- 8 years old (UK Biobank, specific study sample not reported)	UK Biobank -- 54% female (sex breakdown not reported for sample of N=143,685)	N(overall) = 143,685; N(male) = 48,855, N(female) = 59,472	https://biobank.ndph.ox.ac.uk/crystal/field.cgi?id=21003 http://www.nealelab.is/uk-biobank
Daily smoking	Meta-analysis conducted by the GWAS & Sequencing Consortium of Alcohol and Nicotine use (GSCAN)	~100% European ancestry	Not reported, age included as covariate in GWAS	52%	249,752	https://www.ncbi.nlm.nih.gov/pmc/articles/PMC6358542/
Lifetime Smoking	UK Biobank	UK Biobank is ~95% European ancestry (Constantinescu et al., 2022)	58 +/- 8 years old (UK Biobank, specific study sample not reported)	UK Biobank -- 54% female	Pack years of smoking (N=142,387), pack years of smoking as a proportion of life span exposed to smoking (N=142,387), risk of being a light smoker (N=123,894)	https://biobank.ndph.ox.ac.uk/crystal/field.cgi?id=21003<br><br>https://gwas.mrcieu.ac.uk/
Average alcohol consumed on typical drinking day (male)	A Neale Lab analysis of the UK Biobank	UK Biobank is ~95% European ancestry (Constantinescu et al., 2022)	58 +/- 8 years old (UK Biobank, specific study sample not reported)	100% male	48,784	https://biobank.ndph.ox.ac.uk/crystal/field.cgi?id=21003 http://www.nealelab.is/uk-biobank
Average alcohol consumed on typical drinking day (female)	A Neale Lab analysis of the UK Biobank	UK Biobank is ~95% European ancestry (Constantinescu et al., 2022)	58 +/- 8 years old (UK Biobank, specific study sample not reported)	100% female	59,472	https://biobank.ndph.ox.ac.uk/crystal/field.cgi?id=21003 http://www.nealelab.is/uk-biobank

© 2022 Mavromatis LA et al. *JAMA Psychiatry.*

eTable 3. ENIGMA global cortical thickness and surface area instrument SNPs

Exposure	SNP	P-value	Effect allele	Other allele	Beta	SE	Sample size	EAF	R2	F-statistic	95% CI lower	95% CI upper
Global cortical surface area	rs79600142	2.331E-32	T	C	1696.826	143.273	29435	0.780	0.005	140.264	1416.011	1977.641
Global cortical surface area	rs11759026	4.106E-22	A	G	-1301.520	134.615	31907	0.762	0.003	93.478	-1565.367	-1037.674
Global cortical surface area	rs10878349	4.829E-21	A	G	1039.993	110.486	32176	0.490	0.003	88.602	823.440	1256.547
Global cortical surface area	rs34464850	6.758E-16	C	G	1233.185	152.720	31984	0.153	0.002	65.203	933.854	1532.517
Global cortical surface area	rs1628768	1.696E-13	T	C	-972.978	132.004	32176	0.761	0.002	54.328	-1231.708	-714.249
Global cortical surface area	rs2802295	2.543E-10	A	G	-714.585	112.989	32176	0.379	0.001	39.997	-936.045	-493.125
Global cortical surface area	rs12357321	5.217E-09	A	G	-698.745	119.646	32176	0.321	0.001	34.107	-933.252	-464.239
Global cortical surface area	rs12630663	1.27E-08	T	C	-632.810	111.212	32176	0.588	0.001	32.377	-850.787	-414.834
Global cortical surface area	rs10876864	2.43E-08	A	G	-628.590	112.685	31319	0.577	0.001	31.117	-849.454	-407.726
Global cortical surface area	rs2301718	2.547E-08	A	G	737.221	132.355	32176	0.227	0.001	31.025	477.804	996.638
Global cortical surface area	rs7715167	2.653E-08	T	C	-662.753	119.137	32068	0.386	0.001	30.946	-896.263	-429.244
Global cortical surface area	rs386424	4.519E-08	T	G	-656.543	120.042	32176	0.699	0.001	29.913	-891.826	-421.261
Global cortical thickness	rs533577	8.426E-11	T	C	-0.005	0.000	32872	0.494	0.001	39.063	-0.007	-0.003
Global cortical thickness	rs2316766	2.903E-10	T	G	0.006	0.001	26063	0.210	0.002	39.347	0.005	0.009

© 2022 Mavromatis LA et al. *JAMA Psychiatry.*

Global cortical thickness	rs11692435	3.179E-10	A	G	-0.009	0.001	29128	0.091	0.001	36.804	-0.012	-0.006
Global cortical thickness	rs35021943	2.979E-09	A	C	-0.005	0.000	32872	0.758	0.001	32.111	-0.007	-0.003
Global cortical thickness	rs6738528	7.324E-09	A	T	0.004	0.000	32872	0.398	0.001	31.641	0.003	0.006
Global cortical thickness	rs7824177	8.922E-09	A	G	0.005	0.001	32872	0.838	0.001	34.810	0.004	0.008

eTable 36. Leave-one-out MR of GCT on DPW, binge drinking frequency

**Exposure**	**Outcome**	**N**	**Excluded SNP**	**Beta**	**SE**	**P**	**95% CI lower**	**95% CI upper**
Global cortical thickness	Drinks per week	491434	rs11692435	-0.632	0.213	0.003	-1.050	-0.214
Global cortical thickness	Drinks per week	491434	rs35021943	-0.934	0.306	0.002	-1.533	-0.335
Global cortical thickness	Drinks per week	491434	rs533577	-0.886	0.325	0.006	-1.522	-0.250
Global cortical thickness	Drinks per week	491434	rs6738528	-0.900	0.313	0.004	-1.513	-0.286
Global cortical thickness	Drinks per week	491434	rs7824177	-1.030	0.230	0.000	-1.482	-0.578
Global cortical thickness	Drinks per week	491434	None -- IVW/post-LASSO IVW estimate	-0.881	0.247	0.000	-1.365	-0.397
Global cortical thickness	Binge drinking frequency	143445	rs11692435	-1.257	0.536	0.019	-2.308	-0.207
Global cortical thickness	Binge drinking frequency	143445	rs35021943	-1.545	0.647	0.017	-2.814	-0.277
Global cortical thickness	Binge drinking frequency	143445	rs533577	-2.008	0.541	0.000	-3.068	-0.948
Global cortical thickness	Binge drinking frequency	143445	rs6738528	-1.509	0.639	0.018	-2.761	-0.257
Global cortical thickness	Binge drinking frequency	143445	rs7824177	-1.899	0.560	0.001	-2.997	-0.801
Global cortical thickness	Binge drinking frequency	143445	None -- Post-Lasso IVW estimate	-1.643	0.519	0.002	-2.661	-0.625

© 2022 Mavromatis LA et al. *JAMA Psychiatry.*

eTable 42. Unique TWAS-identified genes significant (FDR=0.05) for both cortical thickness, alcohol use behavior(s)

Gene	Type of gene	Associated alcohol use behavior(s)
LRRC37A4P	Psuedogene	Binge
PLEKHM1	Protein Coding Gene	Binge, DPW
LRRC37A2	Protein Coding Gene	Binge, DPW
CRHR1-IT1	Psuedogene	Binge, DPW
CRHR1	Protein Coding Gene	Binge, DPW
ARHGAP27	Protein Coding Gene	Binge, DPW
WNT3	Protein Coding Gene	DPW
LRRC37A	Protein Coding Gene	Binge, DPW
ACTR1B	Protein Coding Gene	DPW
RTN1	Protein Coding Gene	DPW
*All genes associated with alcohol use behavior(s), cortical thickness with opposite directionality. For full details on gene-phenotype associations, cortical reference panels, and gene information, see **eTables 38-41**.		

© 2022 Mavromatis LA et al. *JAMA Psychiatry.*
